# Construction of porous cationic frameworks by crosslinking polyhedral oligomeric silsesquioxane units with N-heterocyclic linkers

**DOI:** 10.1038/srep11236

**Published:** 2015-06-11

**Authors:** Guojian Chen, Yu Zhou, Xiaochen Wang, Jing Li, Shuang Xue, Yangqing Liu, Qian Wang, Jun Wang

**Affiliations:** 1 State Key Laboratory of Materials-Oriented Chemical Engineering, College of Chemistry and Chemical Engineering, Nanjing Tech University, Nanjing 210009, China

## Abstract

In fields of materials science and chemistry, ionic-type porous materials attract increasing attention due to significant ion-exchanging capacity for accessing diversified applications. Facing the fact that porous cationic materials with robust and stable frameworks are very rare, novel tactics that can create new type members are highly desired. Here we report the first family of polyhedral oligomeric silsesquioxane (POSS) based porous cationic frameworks (PCIF-*n*) with enriched poly(ionic liquid)-like cationic structures, tunable mesoporosities, high surface areas (up to 1,025 m^2^ g^−1^) and large pore volumes (up to 0.90 cm^3^ g^−1^). Our strategy is designing the new rigid POSS unit of octakis(chloromethyl)silsesquioxane and reacting it with the rigid N-heterocyclic cross-linkers (typically 4,4′-bipyridine) for preparing the desired porous cationic frameworks. The PCIF-*n* materials possess large surface area, hydrophobic and special anion-exchanging property, and thus are used as the supports for loading guest species PMo_10_V_2_O_40_^5−^; the resultant hybrid behaves as an efficient heterogeneous catalyst for aerobic oxidation of benzene and H_2_O_2_-mediated oxidation of cyclohexane.

**F**unctional porous materials have emerged endlessly, driven by the ever-increasing demand for diverse applications in catalysis, adsorption, separation, electronics, fluorescence, *etc.*^1^,^2^,^3^,^4^. In pioneering works, the skeletons of porous materials can be inorganic, inorganic-organic hybrid and purely organic forms, including zeolites^5^, mesoporous silicas^6^, periodic mesoporous organosilicas (PMOs)^7^, metal-organic frameworks (MOFs)^1^, covalent organic frameworks (COFs)^2^, and porous organic polymers (POPs)^3^. Nowadays, it is a hot topic to facilely transform task-specific building blocks into porous periodic structures with large surface areas and required functionality, wherein the key point is to design new building units and choose suitable synthetic strategy.

Polyhedral oligomeric silsesquioxanes (POSS) are a class of well-defined inorganic silica nanocages surrounded by organic functional groups, which find versatile potential applications in materials science^8^,^9^,^10^. The most intriguing feature for POSS blocks is their three dimensional (3D) inorganic-organic hybrid rigid symmetric structure with exceptional robustness to heat and water, thus ideal for constructing porous inorganic-organic hybrid materials^11^. Recently, various POSS-based micro-/mesoporous networks have been achieved through template-directed co-assembly^12^,^13^ or covalent bond linking of functionalized POSS units via chemical hydrosilation^14^,^15^, coupling reactions (Sonogashira, Heck and Yamamoto)^16^,^17^,^18^,^19^,^20^, Friedel-Crafts reaction^21^,^22^ and polymerization^23^. The applications of those covalently linked POSS-based porous polymers are always limited to the moderate adsorption capability for gases (e.g. H_2_ or CO_2_), mostly owing to the undecorated neutral skeletons. In contrast, the ionic sites within charged networks and the resultant strong electrostatic fields are more accommodable for ionic active species and guest molecules^24^. Therefore, porous ionic solid materials are becoming hot topics in areas of porous crystals^25^, MOFs^26^,^27^,^28^,^29^, polymers^30^,^31^,^32^, nanoparticle networks^33^, PMOs with ionic liquids framework^34^ and mesoporous ionic liquid-polyoxometalate (IL-POM) hybrids^35^,^36^. Such rapid emergence of ionic solid materials is thanks to their fascinating ion-exchange properties and solid-state ionic nanospaces, enabling their applications in heterogeneous catalysis^27^,^28^,^34^,^35^,^36^, gas storage and separation^26^,^30^,^32^, and ionic pollutants adsorption^29^. Nevertheless, up to date, rare porous cationic networks with robust and stable frameworks are reported, and moreover, no such type framework has been fabricated from POSS units because the existing POSS-based porous polymers have neutral skeletons that cannot provide any exchangeable ionic sites.

Herein, a new rigid POSS building block, octakis(chloromethyl)silsesquioxane (ClMePOSS), is designed and applied to construct porous cationic frameworks by reacting with the N-heterocyclic cross-linkers such as 4,4′-bipyridine (4,4′-bpy) (Fig. 1). Ionic liquids (ILs) are a family of liquid organic molten salts frequently prepared from quaternization of N-heterocyclic compounds and alkyl halides^37^. Functional POSS moieties have been used as anions or cations to prepare POSS-based ILs^38^,^39^. Very recently, Leng *et al.*^40^ reported a POSS-derived ionic copolymer by free radical copolymerization of the vinylimidazole-functionalized POSS unit and IL monomer. The copolymer was a solvable oligomer with the surface area of 42.2 m^2^ g^−1^ rather than a high-surface-area robust framework. Nevertheless, no reports have appeared on the direct construction of robust porous cationic frameworks by using POSS building blocks. In our opinion, the vital factor for developing one-step quaternization process towards the desired POSS-based porous cationic framework, though a huge challenge, is to seek a rigid alkyl halide functionalized POSS unit. This motivates the innovative design of rigid ClMePOSS tethered with the specifically tailored chloromethyl groups, providing eight possible positions/directions around one POSS cage to react with the organic linker 4,4′-bpy using nucleophilic substitution reaction. Through this synthetic process, we directly fabricate the novel POSS-based porous cationic framework (named as PCIF-1) with a very high surface area up to 1,025 m^2^ g^−1^ and enriched cationic sites, which can be regarded as a new member of porous ion-exchange materials, breaking through the neutral networks of previous POSS-based porous materials. Various PCIF series materials are achieved by varying the N-heterocyclic organic linkers. The features of PCIF-1 encourage us to broaden its potential application in catalysis by loading bulky anionic POM PMo_10_V_2_O_40_^5−^ (PMoV) through anion-exchange process. The obtained hybrid PMoV@PCIF-1 demonstrates as an efficient and recyclable heterogeneous catalyst for liquid-phase aerobic oxidation of benzene to phenol and H_2_O_2_-mediated oxidation of cyclohexane.

## Results

### Formation of POSS-based porous cationic frameworks

**Figure 1:**
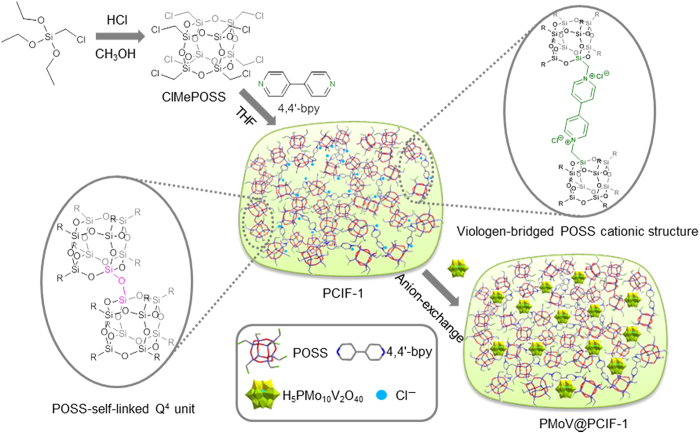
Synthetic procedure of POSS-based porous cationic framework PCIF-1. From original synthesis of a new octakis(chloromethyl)silsesquioxane (ClMePOSS) monomer to the successive quaternization reaction of ClMePOSS with 4,4′-bipyridine (4,4′-bpy) to form PCIF-1. Then, bulky PMo_10_V_2_O_40_^5−^ (PMoV) anions are loaded into PCIF-1 to obtain the PMoV@PCIF-1 catalyst via anion-exchange process.

Fig. 1 shows the synthetic procedure for PCIF-1 produced from the POSS unit and 4,4′-bpy. In the synthesis, screening POSS monomers is the most crucial point for simultaneously getting large surface areas and poly(ionic liquid)-like cationic structures on the POSS-derived frameworks. Therefore, through acid-catalyzed condensation of (chloromethyl)triethoxysilane, we successfully synthesize a new rigid reactive POSS building unit, octakis(chloromethyl)silsesquioxane (ClMePOSS), the structure of which is characterized by ^1^H NMR, ^13^C NMR, ^29^Si NMR and MALDI-TOF MS spectra (Supplementary Figs. S2-S5). As expected, with the catalyst-free solvothermal process in tetrahydrofuran (THF), ClMePOSS is able to react with 4,4′-bpy and gives rise to a gel-state product, finally producing the brittle solid PCIF-1 after drying.

**Figure 2:**
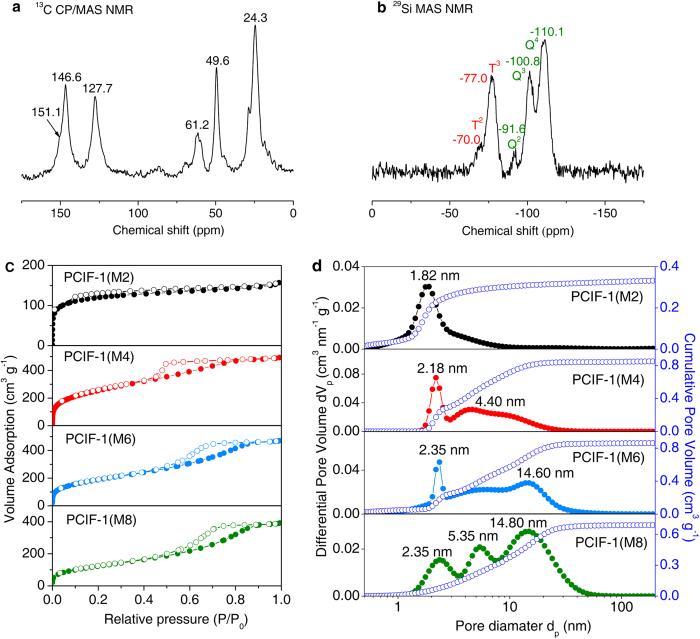
Solid-state NMR spectra and pore structure characterizations. (**a**) ^13^C CP/MAS NMR spectrum of a typical sample of PCIF-1(M4). (**b**) ^29^Si MAS NMR spectrum of PCIF-1(M4). (**c**) N_2_ adsorption-desorption isotherms of PCIF-1(M*x*) series samples with *x* (2, 4, 6, 8) standing for the molar ratio of 4,4′-bpy to ClMePOSS in initial synthesis mixtures. (**d**) Cumulative pore volume and pore size distribution of PCIF-1(M*x*) series calculated by using a slit/cylindrical NLDFT model.

The solid-state ^13^C CP/MAS NMR spectrum for PCIF-1 (Fig. 2a) clearly displays new peaks at chemical shifts (δ) 151.0, 146.6, 127.7 and 49.6 ppm attributable to C_bpy_–C, C_bpy_ = N, C_bpy_ = C and SiCH_2_N, respectively, suggesting the formation of Si–C–N bond due to the covalent linkage of 4,4′-bpy and ClMePOSS through the quaternization reaction. The peak at 24.3 ppm is due to the residual end group in ClMePOSS. The solid-state ^29^Si MAS NMR spectra (Fig. 2b) reveals two chemical shifts at around δ = −70.0 and −77.0 ppm attributable to the retentive T^2^ and T^3^ units (T^*n*^: CSi(OSi)^*n*^(OH)^3−*n*^), confirming the presence of cubic POSS cage structures^8^, in accordance with previous POSS-based porous polymers^17^,^18^. The T^2^ unit may be directly from the minor T^2^ structure in POSS monomer and/or arise from partial collapse of POSS cages, which has been observed in the syntheses of POSS-based materials^17^,^18^,^19^,^20^,^21^. In addition, the three peaks featured for the framework sites in siliceous structures Si(OSi)_4_ (Q^4^), HOSi(OSi)_3_ (Q^3^), and (HO)_2_Si(OSi)_2_ (Q^2^) appear explicitly at −110.1, −100.8, and −91.6 ppm, respectively^34^. Q^4^ structure should be resulted by the self-condensation of silanols from the formed Q^3^ units due to the Si–C bonds cleavage of T^3^ units^20^. Minor Q^2^ structure of silicons may be derived from the cleavage of Si–C in original T^2^ units or the decomposition of siloxane bond in Q^3^ units. Previous studies have shown two possible reasons for the destruction/distortion of POSS cage; one is owning to the basic N-functional group that would break the Si–O and Si–C bonds^20^; the other is ascribed to the distortion of cubic POSS cages when they are connected by rigid linkers^17^,^19^,^20^. It is well known that Si–C bond is strong and thermally stable, however, its stability can be dramatically affected by the introduction of electronegative substituent groups (e.g. chlorine, χ (Cl) = 3.16) attached to α carbon adjacent to Si atom^41^. Besides, the attack of nucleophilic reagents will promote the cleavage of Si–C bonds. To obtain the desired cationic porous networks, we design the very rigid ClMePOSS and select the rigid cross-linker 4,4′-bpy, which exacerbate the distortion of incorporated POSS cages so as to reduce the structural constraints or stresses in the formed networks. Meanwhile, during the quaternization reaction, the nucleophilic reagent 4,4′-bpy is also contributed to the partial cleavage of Si–C bonds, causing the formation of Si–OH of Q^3^ silicons in the present of the inevitable water^41^,^42^. It is thus understandable that a large number of Q^3^ units are formed in our synthesis and further self-condensed to give the Q^4^ structure. In other words, many POSS units are self-linked together covalently by the siloxane linkages as well as cross-linked by the 4,4′-bpy linkers in PCIF-1 framework. The process implies that the POSS cages remain essentially intact inside the formed net-framework. Indeed, there are two main POSS building units, *i.e.*, the original T^3^ POSS unit and the derivative Q^4^-structured POSS unit, and both of them can be bridged by the cross-linker 4,4′-bpy to together form the PCIF-1 network (see the schematic structures in Fig. 1 and Supplementary Fig. S8). The possible destruction of POSS cages on the weak basic N sites can be excluded by the separately conducted control measurements, in which we employ 2,2′-bipyridine, pyrazine, pyridine or 1-methylimidazole (Supplementary Fig. S9) to react with ClMePOSS and only obtain water solvable POSS-IL products (*i.e.*, no siliceous precipitation is observed), since there is only one N atom for each of the four counterparts that can be quaternized by the –CH_2_Cl group on POSS skeleton. These control measurements plus the NMR analysis results finally confirm that the attack of nucleophilic rigid organic linker and the distortion of POSS cages accelerate the formation of Si–C–N bonds and partial cleavage Si–C bonds, forming PCIF-1 with both T^n^ and Q^n^ silicons units.

A series of solid products PCIF-1(M*x*) are obtained by varying the molar ratio of 4,4′-bpy to ClMePOSS in the initial synthesis mixtures (denoted as *x*). The N_2_ sorption results for PCIF-1(M2, M4, M6, M8) series exhibit type IV isotherms with a sharp uptake at low relative pressures and a slowly increasing one at higher relative pressures (Fig. 2c), indicative of typical micro-/mesoporous materials. With the increase of the 4,4′-bpy amount, the H1 type hysteresis loop gradually shifts to the higher relative pressure, suggesting expansion of mesopores size. Fig. 2d displays the results of the cumulative pore volume and pore size distributions. All the PCIF-1 samples have narrow distributions around ~2.0 nm, the down limit of mesopores; meanwhile, relatively broad distributions appear in the range of larger mesopores, especially for those samples with higher amounts of 4,4′-bpy. Cumulative pore volume analysis results obviously exhibit 85% of the pore volumes for PCIF-1(M4, M6, M8) are contributed by the mesopores (2 ~ 15 nm), while 68% of the pore volume for PCIF-1(M2) originates from the super-micropores around 1.8 nm. Further, mesoporosities with narrower distributions centered at 3.7 nm for PCIF-1(M4) and 5.4 nm for PCIF-1(M6, M8) are determined by Barrett-Joyner-Halenda (BJH) method (Supplementary Fig. S10). The existence of enriched small pore sizes for PCIF-1(M2) is also reflected by the obvious declined curve with a weaker shoulder at 3.7 nm. Table 1 lists the textural properties and N contents (wt%) of PCIF-1 series. As seen, with increasing molar ratios of 4,4′-bpy to ClMePOSS from *x* = 2 to *x* = 8 in synthesis menus, N contents in final products increase continuously from 0.24 wt% to 1.59 wt%, implying the formation of more 4,4′-bpy-linked POSS structures. On the other hand, as *x* increases, the average pore size continuously increases from 2.16 to 5.24 nm, while the surface areas (S_BET_) and total pore volume (V_total_) firstly increase and then decrease, giving the maximum surface area (942 m^2^ g^−1^) and pore volume (0.76 cm^3^ g^−1^) at *x* = 4. Moreover, elemental synthetic parameters are investigated. Typical syntheses in this work are carried out with THF as solvent because of its well dissolvability to reactants. Other solvents are also attempted, but unsuccessful due to immiscibility with the POSS monomer. Toluene can dissolve the reactants but only produces the low surface area of 38 m^2^ g^−1^ and pore volume of 0.28 cm^3^ g^−1^ (Supplementary Fig. S11). By adjusting the key conditions (reaction time, reaction temperature and THF amount) during the synthesis of PCIF-1(M4) sample, the textural properties (the surface area, pore volume and average pore size) can be facilely controlled in large range while keeping the similar pore shape (Supplementary Tables S1-S3 and Figs S12-S15), and thus provide the highest surface area of 1,025 m^2^ g^−1^ and pore volume of 0.90 cm^3^ g^−1^.

For the purpose to further understand the formation process of porous cationic frameworks and diversify potential functionalities, the POSS monomer and N-bearing organic cross-linkers are varied in the synthesis. When octakis(3-chloropropyl)silsesquioxane (ClPrPOSS)^43^(Supplementary Fig. S1) with similar structure as ClMePOSS but softer chloropropyl groups, is used as the monomer to react with 4,4′-bpy, the obtained material shows a very low surface area of 7 m^2^ g^−1^, confirming the importance of the rigid POSS units. Also apart from 4,4′-bpy, various N-bearing organic cross-linkers with different rigidities, such as 1,2-bis(4-pyridyl)ethylene (bpe), 1,2-bis(4-pyridyl)ethane (bpea), 1,3-bis(4-pyridyl)propane (bppa), bis(1-imidazolyl)methane (bim), N,N,N’,N’-tetramethylethylenediamine (tmeda) and 1,4-diazabicyclo[2.2.2]octane (dabco) (Table 2), are employed to react with ClMePOSS, forming the PCIF-*n* series hybrids (*n* corresponds to the organic linkers; *n* = 1: 4,4′-bpy; *n* = 2 ~ 7: the above six linkers sequentially). N_2_ sorption isotherms and pore size distribution curves for PCIF-*n* series (*n* = 2 ~ 7) demonstrate the formation of hierarchically porous materials (Supplementary Fig. S16) with considerable amounts of N contents (Table 2). When the four pyridine-based linkers are regarded (Table 2, PCIF-1 ~ 4), one can see that the most rigid molecule 4,4′-bpy shows the highest surface area of 1,025 m^2^ g^−1^, and the less rigid bpe leads to a remarkably lowered surface area of 396 m^2^ g^−1^ (though still a considerably high value); on the contrary, the two flexible molecules bppa and bpea only cause very low surface areas ≤ 40 m^2^ g^−1^. Above comparisons allow to conclude that the rigid structures of both POSS monomer and N-bearing organic cross-linkers are prerequisites for creating POSS-based porous cationic polymeric frameworks. The proposal is confirmed by another three rigid cross-linkers of non-pyridine-based N-bearing organic molecules (*i.e.* bim, tmeda and dabco), with which considerably high surface areas of 183 ~ 729 m^2^ g^−1^ and micro-/mesoporous framework structures are achieved (Supplementary Fig. S16 and Table 2, PCIF-5 ~ 7).

### Further characterizations of PCIF-1

**Figure 3:**
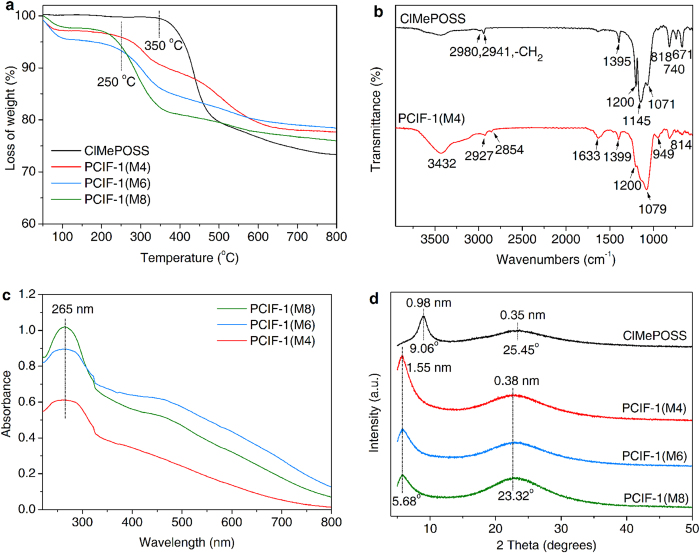
Further structure characterizations of PCIF-1. (**a**) TGA curves of PCIF-1 series. (**b**) FT-IR spectra of ClMePOSS and PCIF-1(M4). (**c**) UV-vis absorption spectra of PCIF-1 series. (**d**) XRD patterns of ClMePOSS and PCIF-1 series.

The thermogravimetric analysis (TGA) for ClMePOSS shows that it is thermally stable up to 350 ^o^C, while the decomposition takes place at *ca.* 250 ^o^C for PCIF-1 networks (Fig. 3a). This phenomenon implies the formation of cationic networks from covalent POSS molecules, considering that the thermal stability for ionic covalent bonds is often inferior to that of covalent linkages. Similar results have been observed in such previous cationic materials as IL-like silica networks^33^ and positively charged porous aromatic frameworks^30^. Figure 3b compares the FT-IR spectra of ClMePOSS and PCIF-1(M4). The cubic silsesquioxane cage in ClMePOSS monomer is demonstrated by the strong peak at 1,145 cm^−1^ for the typical Si–O–Si asymmetric stretching vibration^8^. The specially designed reactive chloromethyl (–CH_2_Cl) tethered on POSS skeleton is clearly characterized by the wagging vibration band at 1,200 cm^−1,^^21^, confirmed by the adjunctive Si–CH_2_ band in-plane deformation (1,395 cm^−1^), CH_2_ stretching vibrations (2,980 and 2,941 cm^−1^), Si–C stretching (818 cm^−1^) and C–Cl stretching vibrations (740 and 671 cm^−1^). After bridged with 4,4′-bpy, the resulting hybrid PCIF-1(M4) retains Si–O–Si structure with the feature band slightly shifted to the lower wavenumber of 1,079 cm^−1^, due to the spatial distortion of POSS cages^8^. The new band at 1,633 cm^−1^ with moderate intensity can be attributed to the C = N stretching vibration originated from 4,4′-bpy organic moiety. The bands at 3,432 and 949 cm^−1^ are due to the C–H and Si–O stretching vibrations derived from silanols of Q^n^ units. For selected PCIF-1 samples, on the other hand, UV-visible (UV-vis) absorption spectra (Fig. 3c) illustrate an intense band at 265 nm ascribed to the π-π* intramolecular transitions of the bipyridinium (also called viologen) central moiety^44^, characteristic of typical organic-inorganic hybrid structure. All the spectral characterizations evidence once again the formation of viologen-bridged PCIF-1 via the reaction of ClMePOSS and 4,4′-bpy, as drawn from the NMR results.

The X-ray diffraction (XRD) patterns (Fig. 3d) indicate that the obtained POSS-based porous cationic networks are not fully-crystallized. However, compared with many other amorphous porous POSS polymers, the present XRD patterns show a sharp Bragg diffraction peak at 5.68° and a broad one around 23.32^o^ with the corresponding *d*-spacing values of 1.55 nm and 0.38 nm, attributable to the length of functionalized POSS units and the siloxane bonds of POSS cores, respectively^8^. This result implies that PCIF-1 possesses a molecular-level periodic arrangement with moderate long range order^18^. In particular, the low angle of 5.68^o^ for PCIF-1 is obviously lower than the one (9.06^o^) for the semi-crystalline ClMePOSS, indicative of increasing *d*-spacing values from 0.98 nm to 1.55 nm, which is attributed to the enhanced molecular dimension among POSS cages (*ca.* 1.0 nm) linked by the rigid organic moiety 4,4′-bpy^8^.

**Figure 4:**
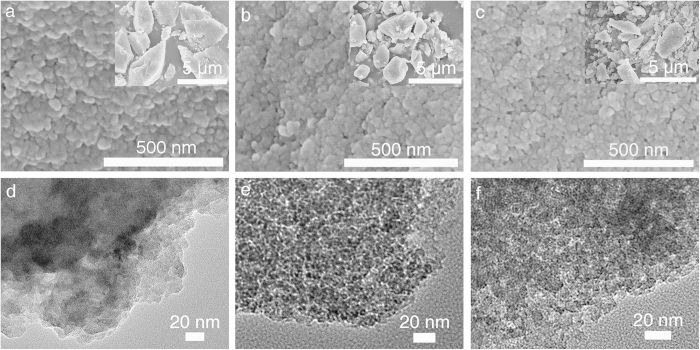
SEM images and HR-TEM images of PCIF-1 series. (**a**, **d**) PCIF-1(M4), (**b**, **e**) PCIF-1(M6) and (**c**, **f**) PCIF-1(M8).

Scanning electron microscopy (SEM) images indicate that all the PCIF-1 series are micrometer-scale irregular blocks with fluffy rough surfaces composed of aggregated nanoparticles with the sizes of 20 ~ 30 nm (Figs 4a~c). The observable mesopores in SEM images arise from nanovoids among the closely packed nanoparticles. Same samples are further examined by the high resolution transmission electron microscopy (HR-TEM) images (Figs 4d~f), showing that PCIF-1(M4) has abundant small mesopores, and PCIF-1(M6, M8) possess somewhat uniform worm-like larger mesopores with sizes of 3 ~ 6 nm, well consistent with the results of pore size distributions measured by N_2_ sorption.

Moreover, the stability of the POSS-based porous materials in different organic solvents and aqueous solution is examined over one typical example PCIF-1(M4). After soaked in water and various organic solvents (such as CH_3_OH, CHCl_3_, DMF, and DMSO) for one day, the solid is collected by filtration, washed, dried and then characterized by N_2_ sorption, SEM, XRD, and FT-IR. Almost the same results of the above characterizations (Supplementary Figs. S17-S20 and Table S4) are observed over the treated sample as the fresh one, demonstrating well stability of PCIF samples in common solvents.

### CO_2_ adsorption capability

In recent years, porous polymer materials are a typical kind of solid adsorbents for CO_2_ capture^45^, and thus CO_2_ adsorption measurements are conducted on the selected samples of PCIF-1(M4, M6, M8) that own different surface areas. The CO_2_ uptakes of PCIF-1(M4) with the highest surface area are 0.96 mmol g^−1^ (4.22 wt%, at 273 K, 1 bar) and 0.68 mmol g^−1^ (2.99 wt%, at 298 K, 1 bar) (Supplementary Fig. S21), comparable to the reported results of some MOF^46^, POP^47^ and POSS-based porous polymers with more or less similar surface areas^19^,^20^,^22^. The CO_2_ uptakes of PCIF-1(M6) and PCIF-1(M8) decrease to 0.60 mmol g^−1^ and 0.47 mmol g^−1^ (298 K, 1 bar), respectively, mostly due to the lowered surface areas. The relative low CO_2_ uptakes can be assigned to (i) their enriched mesopores but with low proportion of microporosity; (ii) lacking of sufficient basic sites to provide enough driving force for the adsorption of CO_2_^45^,^46^. The above phenomena also suggest that the cationic sites present a weak direct advantage for CO_2_ capture, which may provide some clues to design more efficient CO_2_ adsorbents after further functionalizing this cationic framework. Therefore, in this stage, we focus on the application of PCIF-1 as porous anion-exchangers and catalyst supports, so as to give full play to the advantage of cationic framework with large surface area.

### Anion-exchange property

**Figure 5:**
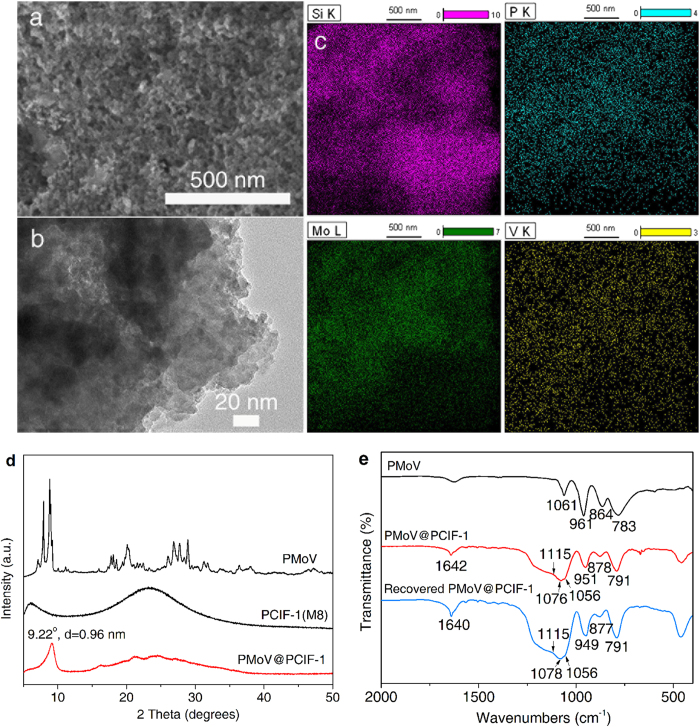
Morphology, elemental distribution and characterizations of PMoV@PCIF-1 . (**a**) SEM image. (**b**) TEM image. (**c**) EDS elemental mapping of Si, P, Mo, V elements. (**d**) XRD patterns. (**e**) FT-IR spectra.

Taking into account of the cationic porous polymeric skeletons with the abundant mesoporosity for PCIF-1 series, one may expect this kind of materials to be excellent anion-exchangers, even with high capability to exchange large sized anions. Owing to the considerably high surface area (460 m^2^ g^−1^) and the highest content of N cationic center (1.59 wt%, 1.14 mmol g^−1^), herein PCIF-1(M8) is selected for evaluating the anion-exchange capability. The bulky PMo_10_V_2_O_40_^5−^ (PMoV) with the size of ~1.0 nm is chosen as guest counter anion. Based on the principle of ion-exchanging, the driving force for the anion-exchange process is the differentiation of concentrations of multivalent PMoV anions between the ethanol solution and the bulk phase of the cationic framework (Supplementary Fig. S22)^48^. The higher ion-pairing energy between the multivalent PMoV anion and the N cationic center with respect to the Cl^−^-resulted ion-pair also facilitates the formation of PMoV@PCIF-1^48^. SEM and TEM images for the resulting PMoV@PCIF-1 (Figs 5a,b) show a similar micro-morphology as its parent PCIF-1(M8) with well-preserved mesoporous structure. Elemental mapping analyses with energy-dispersive X-ray spectrometry (EDS) for Si, P, Mo and V elements (Fig. 5c) indicate the successful ion-exchange of PMoV anions with a homogeneous distribution. The organic elements (C, H, N, O) and inorganic elements (Si, P, Mo and V) of PMoV@PCIF-1 are measured by CHN elemental analysis and ICP-AES respectively, giving the following composition (wt%): C, 7.71; H, 2.30; N, 0.96; O, 36.58; Si, 25.99; P, 0.42; Mo, 6.54; V, 0.70. Therefore, the mass content of PMoV is 11.5 wt% (0.066 mmol g^−1^) in the hybrid sample, which is confirmed by the TGA analyses (Supplementary Fig. S23). The ion exchange degree is *ca.* 30% calculated from the contents of N ionic centers in PCIF-1(M8) and PMoV anions in PMoV@PCIF-1. The residue rate of chloride ions is *ca.* 1.81 wt% and 1.18 at% (atomic concentration) measured by EDS and X-ray photoelectron spectroscopy (XPS) analyses, respectively (Supplementary Fig. S24 and S25), suggesting a low residue level of chloride in the catalyst. The surface area, pore volume and most probable pore size of PMoV@PCIF-1 are 413 m^2^ g^−1^, 0.54 cm^3^ g^−1^ and 3.7 nm, respectively (Supplementary Fig. S26), slightly smaller than the parent PCIF-1(M8), owing to that the incorporated POM-anions partially occupies the framework nanospaces. Compared with the crystalline parent H_5_PMo_10_V_2_O_40_, a new XRD peak appears at 2θ = 9.22° (*d*-spacing value of 0.96 nm, Fig. 5d), indicating the well-organized distribution of POM clusters onto the load points of the POSS-based cationic framework^35^,^36^. The FT-IR spectrum of PMoV@PCIF-1 (Fig. 5e) gives a set of characteristic bands for Keggin structure at 1,076 and 1,056 cm^−1^ ν(P-O_a_), 951 cm^−1^ ν(M-O_b_-M) (M = Mo or V), 878 cm^−1^ ν(M-O_c_-M) and 791 cm^−1^ ν(M-O_d_)^49^, further proving the presence of PMoV in the PCIF-1 network. These bands occur with slight shifts in contrast to pure PMoV. The vibration for central oxygen-bonded P-O_a_ branches from a single one at 1,061 cm^−1^ into the two bands at 1,076 and 1,056 cm^−1,^^49^, which is also reflected by the XPS spectra of split P2p peaks at 132.8 and 133.4 eV (Supplementary Fig. S25f). All of these variations reveal strong electronic interactions between framework-cationic sites and POM-anions.

## Discussion

**Figure 6:**
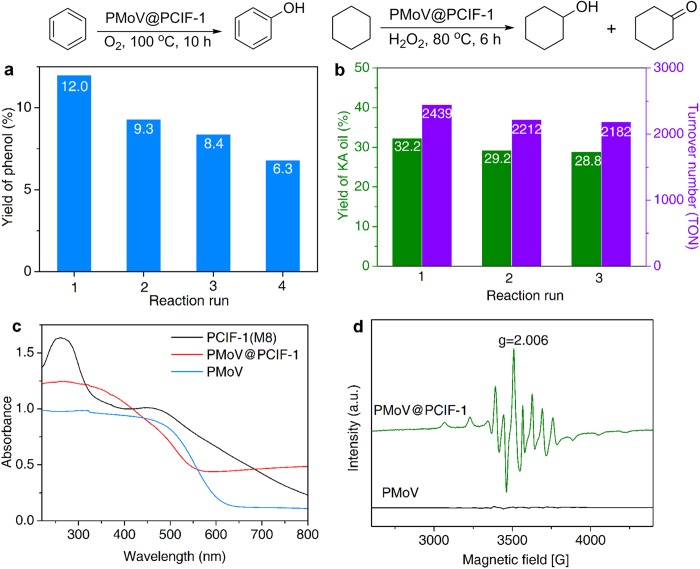
Catalytic oxidation performance and electronic behavior of PMoV@PCIF-1. (**a**) Catalytic performance (yield of phenol) and four-run reusability of PMoV@PCIF-1 for the hydroxylation of benzene with O_2_. (**b**) Catalytic performance (yield of KA oil, TON value) and three-run reusability of PMoV@PCIF-1 for the oxidation of cyclohexane to cyclohexanol and cyclohexanone with H_2_O_2_. (**c**) UV-vis spectra. (**d**) X-band ESR spectra.

The fascinating features of PCIF materials, such as POSS-derived rigid cationic matrix, large surface areas and enriched mesopores, allow accessing new applications by introducing various functional guest anionic species via anion-exchange. POMs are a class of anionic metal-oxide clusters with catalytic redox functionality, and have been used as the catalysts for numerous organic reactions^50^. For example, the V-containing POM-anion PMoV is a well-known catalyst for many oxidation reactions^51^,^52^. However, the practical applications of POM catalysts are often limited by the difficulty in catalyst isolation owing to the high solubility in polar media. As demonstrated above, PMoV anions have been facilely immobilized into the cationic network of PCIF-1 through anion-exchange, which encourages us to attempt the resulted hybrid PMoV@PCIF-1 as the heterogeneous catalyst for organic oxidations. The catalytic performance of PMoV@PCIF-1 is first evaluated in liquid-phase aerobic hydroxylation of benzene to phenol with molecular oxygen O_2_ as the oxidant and ascorbic acid as the reductant, which is potentially very important in petrochemical industry aiming to replace the traditional multi-step cumene process with unsafety and heavy pollution^51^. As seen in Fig. 6a, the high phenol yield of 12.0% is obtained, giving high turnover number (TON) of 136 based on the content of heteropolyanion PMoV. Besides, the equivalent pure PMoV showed a phenol yield of 4.6% (Supplementary Table S5), much lower than that over porous PMoV@PCIF-1 (12.0%), suggesting that dispersing PMoV species on PCIF-1 cationic sites can significantly improve the catalytic performance.

After reaction, the catalyst is conveniently isolated by filtration and reused in the next run. The yields of phenol for the four-run recycling test are 12.0/9.3/8.4/6.8% sequentially (Fig. 6a). The hydroxylation of benzene with O_2_ is very challengeable using a solid material as a heterogeneous catalyst under the harsh reaction conditions. Up to now, the reported heterogeneous catalysts rarely exhibited both high phenol yield and well reusability. Supplementary Table S6 listed the phenol yields in catalyst recycling tests over various heterogeneous catalysts published previously, with the similar aerobic reaction system to our work. Based on the above comparison, one can see that the four-run cycle performance of 12.0/9.3/8.4/6.8% over PMoV@PCIF-1 is superior to the previously reported heterogeneous catalytic systems, presenting both relative high initial phenol yield and well recycle performance at the current research stage^53^,^54^,^55^,^56^,^57^. FT-IR spectrum of the recovered catalyst (Fig. 5e) reveals basically similar structure over the fresh one, therefore, the slow decrease in yields still mostly arises from the contamination of active sites by the deep-oxidation products of tars, which is however currently unavoidable because the target product phenol is actually much easier to be oxidized than the substrate benzene^51^. More importantly, to the best of our knowledge, the TON value (136) is much higher than all the previous V-POM-based catalysts for homogeneous or heterogeneous aerobic oxidation of benzene to phenol^51^,^53^,^55^,^56^,^57^, further illustrating the high activity of the porous catalyst PMoV@PCIF-1.

The high catalytic efficiency of PMoV@PCIF-1 can be attributed to the unique POSS-based cationic framework. On the one hand, the catalyst enables the high dispersion and well accessibility of the catalytically active PMoV anions on the cationic sites of the large-surface-area network matrix with a “point-to-point” manner through strong electrostatic interaction. On the other hand, the incorporated abundant hydrophobic POSS units can dramatically improve the compatibility of the catalyst to hydrophobic organic substrates, benefiting the rapid adsorption of oleophilic benzene and timely release of hydrophilic phenol once produced. Additionally, π-extended viologen cations may influence the electronic state of PMoV anions, and even lead to the occurrence of reduced V^4+^ state. The UV-vis spectroscopy (Fig. 6c) for PMoV@PCIF-1 shows an obvious broad adsorption band at 600 ~ 800 nm assigned to the reduced state of V^4+^ species in Keggin structure^58^, which is also demonstrated by the typical eight fold hyperfine splitting signal centered at G = 3,500 (with a *g* value of 2.006) in the electron spin resonance (ESR) spectrum (Fig. 6d)^58^. XPS spectra of the V2p_3/2_ peak further confirms the existence of two V valence states at 517.0 and 516.3 eV for V^5+^ and V^4+^ species^54^, respectively (Supplementary Fig. S25i). By contrast, pure PMoV only contains V^5+^ species, assorting with the silent response in the same regions of its UV-vis and ESR spectra. Therefore, in the view of the already recognized mechanism for aerobic hydroxylation of benzene, the viologen cations on the PCIF-1 network may facilitate and stabilize the V^4+^ -involving ascorbic acid-PMoV intermediate, which activate O_2_ to create the active oxygen species and promote the formation of the final product phenol^56^.

The catalytic performance of PMoV@PCIF-1 is further investigated in H_2_O_2_-mediated oxidation of cyclohexane (a more hydrophobic and inert substrate than benzene) to cyclohexanol and cyclohexanone (KA oil), which is of great significance in industrial concerns. The catalyst exhibits a high KA oil yield of 32.2%, ultra-high TON of 2,439 and can be reused three times without significant loss of activity (Fig. 6b). The achieved high yield and TON are superior to those of many POMs or vanadium-catalyzed catalytic reaction systems for this reaction^59^, and even higher than the data (KA oil yield of 27.2% and TON of 2,060) of the equivalent pure PMoV-catalyzed homogeneous system, further revealing the well performance of PMoV@PCIF-1 catalyst.

Our findings develop a new strategy and direction in creating robust porous cationic framework materials. This work describes the first successful synthesis of PCIF series from one-pot quaternization between the newly designed chloromethyl-POSS unit and N-heterocyclic organic linkers. The obtained cationic frameworks possess tunable large surface areas, hierarchical micro-/mesopores, cationic sites on polymeric matrix with ion-exchangeable various guest counter-anions. By virtue of the enriched cationic sites and narrow distributed mesopores for PCIF-1, the large sized and catalytically active POM-anionic species PMo_10_V_2_O_40_^5−^ (PMoV) has been well immobilized into the cationic framework through anion-exchange. The produced hybrid PMoV@PCIF-1 behaves as a highly efficient heterogeneous catalyst in the two important liquid-phase organic oxidation reactions, *i.e.*, aerobic oxidation of benzene to phenol and H_2_O_2_-based oxidation of cyclohexane to KA oil. We predict that such porous cationic frameworks may facilitate the rapid emergence of more diverse porous ionic materials, even including well-defined crystalline porous ionic frameworks, as well as open up potential applications in wide heterogeneous catalysis, ionic pollutants adsorption, and luminescent materials science.

## Methods

### Materials

(Chloromethyl)triethoxysilane and (3-chloropropyl)trimethoxysilane were purchased from Beijing HWRK Chem. Bis(1-imidazolyl)methane was prepared according to the literature method^60^. All other N-heterocycles 4,4′-bipyridine, 1,2-bis(4-pyridyl)ethene, 1,2-bis(4-pyridyl)ethane, 1,3-bis(4-pyridyl)propane, N,N,N’,N’-tetramethylethylenediamine, 1,4-diazabicyclo[2.2.2]octane, 2,2′-bipyridine, pyrazine, pyridine and 1-methylimidazole were commercially available and used as received.

### Characterization

Fourier transform infrared spectroscopy (FT-IR) was recorded on a Nicolet iS10 FT-IR instrument (KBr discs) in the region 4,000-400 cm^−1^. Solid UV-visible adsorption spectra were measured with a SHIMADZU UV-2600 spectrometer and BaSO_4_ was used as an internal standard. Electron spin resonance (ESR) spectra were recorded on a Bruker EMX-10/12 spectrometer at the X-band at ambient temperature. X-ray photoelectron spectra (XPS) were conducted on a PHI 5000 Versa Probe X-ray photoelectron spectrometer equipped with Al Kα radiation (1486.6 eV). Liquid-state ^1^H NMR and ^13^C NMR spectra were measured with a Bruker DPX500 spectrometer at ambient temperature using TMS as internal reference. Solid-state ^29^Si MAS NMR, ^13^C and ^1^H spin-echo pulse NMR spectra experiments were performed on a Bruker Avance III spectrometer in a magnetic field strength of 9.4 T. The matrix-assisted laser desorption ionization-time of fight mass spectrometry (MALDI-TOF MS) analysis was performed using the Bruker Autoflex mass spectrometer with 2,5-dihydroxybenzoic acid (DHB) as the matrix under positive ion mode. The CHN elemental analysis was performed on an elemental analyzer Vario EL cube. Inorganic chemical compositions of samples were obtained using a Jarrell-Ash1100 inductively coupled plasma atomic emission spectrometry (ICP-AES). Thermogravimetric analysis (TGA) was carried out with a STA409 instrument in nitrogen or air atmosphere at a heating rate of 10 ^o^C min^−1^. X-ray diffraction (XRD) patterns were collected with a SmartLab diffractometer (Rigaku Corporation) equipped with a 9 kW rotating anode Cu source at 40 kV and 200 mA, from 5° to 50° with a scan rate of 0.2° s^−1^. Field emission scanning electron microscope (FESEM; Hitachi S-4800, accelerated voltage: 5 kV) accompanied by Energy dispersive X-ray spectrometry (EDS; accelerated voltage: 20 kV) was used to study the morphology and the elements distribution. Transmission Electron Microscopy (TEM) images were obtained by using a JEOL JEM-2100F 200 kV field-emission transmission electron microscope. N_2_ adsorption isotherms were measured at 77 K using a BELSORP-MINI analyzer and the samples were degassed at 150 °C for 3 h in high vacuum before analysis. The CO_2_ adsorption experiments were measured at 273 K or 298 K under atmospheric pressure (1.0 bar) with a Micrometrics ASAP 2020 automatism isothermal adsorption instrument. Prior to the measurements, the samples were degassed at 150 ^o^C for 12 h.

### Synthesis

First, the POSS monomer octakis(chloromethyl)silsesquioxane (ClMePOSS, Fig. 1) was synthesized as follows. A solution of 150 mL of dry methanol and 5 mL of concentrated hydrochloric acid was placed in a round-bottomed flask, and then (chloromethyl)triethoxysilane (15 g) was slowly added into the solution. The reaction mixture within the closely sealed flask was stirred in a 40 ^o^C constant temperature water bath for two weeks. After the reaction, the solvent was removed by reduced pressure distillation, and dried under vacuum. A white crystalline product ClMePOSS was finally obtained with a yield of 90% (7.0 g). Spectroscopic data of ClMePOSS are as follows (Supplementary Figs. S2-S4). ^1^H NMR (500 MHz, CDCl_3_) δ 2.91 ppm (SiCH_2_). ^13^C NMR (125 MHz, CDCl_3_) δ 24.3 ppm (SiCH_2_). ^29^Si MAS NMR (80 MHz) δ -78.3 ppm (Si-O-Si). MALDI-TOF MS (DHB as matrix, THF as solvent): calcd for [C_8_H_16_O_12_Si_8_Cl_8_] 812.48; found m/z 813.48 [M + H]^+^ (Supplementary Fig. S5). Besides, another POSS monomer octakis(3-chloropropyl)silsesquioxane (ClPrPOSS, Supplementary Fig. S1) was synthesized according to previously reported method^42^(the detailed experiment and the characterization results were shown in the Supplementary Information).

Second, the porous cationic framework was synthesized through the quaternization reaction between the POSS monomer and the organic cross-linker. Typically, ClMePOSS (0.50 g, 0.62 mmol) and 4,4′-bpy (0.39 g, 2.48 mmol) were dissolved in THF (10 mL), and then the solution was moved into a Teflon-lined autoclave, which was taken place at 100 ^o^C in a constant temperature oven for 48 h. After reaction, a gel-state monolithic solid was obtained and then was cut into pieces with stirred in ethanol solution for two hours. The well-dispersed ionic gel ethanol suspension was filtered, thoroughly washed with ethanol, THF, and water, respectively. Finally, the bulk solid was dried in a vacuum at 80 °C to give the product with the solid yield of 45%. The samples prepared using 4,4′-bpy as the cross-linker were named as PCIF-1(M*x*), in which *x* denoted as the molar ratio of 4,4′-bpy to POSS monomer. Other porous ionic hybrid samples including PCIF-2, 3, 4, 5, 6 and 7 were successively prepared by the quaternization reaction of ClMePOSS and N-bearing organic cross-linkers including bpe, bpea, bppa, bim, tmeda and dabco, according to the above described process. Besides, 2,2′-bipyridine, pyrazine, pyridine and 1-methylimidazole were also used to react with ClMePOSS under the same reaction conditions.

At last, the porous hybrid catalyst PMoV@PCIF-1 was prepared by immobilizing vanadium (V)-substituted POM H_5_PMo_10_V_2_O_40_ (PMoV) on PCIF-1 via the anion-exchanging process. The solid PCIF-1(M8) (1.0 g) was first dispersed in ethanol (100 mL) solution, and then the ethanol solution (50 mL) of pure PMoV (0.78 g, 0.45 mmol) was slowly added into the gel-like ethanol solution, following with stirred for 72 h at room temperature. The yellow solid PMoV@PCIF-1 was obtained by the consecutive basic operations including filtration, washing and drying.

### Catalytic tests

The hydroxylation of benzene with O_2_ was carried out in an automatic temperature controllable pressured titanium reactor (100 mL) equipped with a mechanical stirrer. In a typical run, benzene (2.0 mL), catalyst PMoV@PCIF-1 (0.30 g) and ascorbic acid (0.80 g) were successively added into 25 mL of the aqueous solution of acetic acid (50 vol%). Then the system was charged with 2.0 MPa O_2_ at room temperature, the reaction was carried out at 100 ^o^C for 10 h with vigorous stirring. After the reaction, 1,4-dioxane was added into the product mixture as an internal standard for product analysis. The mixture was analyzed by GC (Agilent GC 7890B) equipped with a hydrogen flame ionization detector and capillary column (HP-5, 30 m × 0.25 mm × 0.25 μm). Under the reaction conditions, phenol was the only product detected by GC, and the common by-products (catechol, hydroquinone and benzoquinone) were not found. After the first run of the test, the solid catalyst was recovered from the reaction mixture by centrifugation, washing with ethanol and drying in vacuum oven at 80 ^o^C for 12 h. Then the recovered catalyst was reused in the next run without adding fresh one.

The oxidation of cyclohexane with H_2_O_2_ was tested in a parallel reactor with magnetic stirrers. In a typical experiment, cyclohexane (5 mmol), catalyst PMoV@PCIF-1 (10 mg), acid additive 2,3-pyrazinedicarboxylic (0.3 mmol) and solvent acetonitrile (3 mL) were successively added into the Schlenck tube and the mixture was stirred at 80 ^o^C for several minutes, and then 30 wt% H_2_O_2_ (10 mmol) was added drop by drop using an injection syringe. The reaction mixture in sealed tube was continued for 6 h with heating at 80 ^o^C with reflux and stirring. After the reaction, toluene (2.5 mmol) was added into the mixture as the internal standard, and an excess triphenylphosphine was also added for reducing the formed cyclohexylhydroperoxide to cyclohexanol. Before analysis, the above mixture was diluted by the used solvent acetonitrile and was thoroughly stirred for a homogeneous mixture, which was then analyzed by GC. The solid catalyst also could be recovered by centrifugation, washing with ethanol and vacuum drying, and then directly used for the next run.

## Additional Information

**How to cite this article**: Chen, G. *et al.* Construction of porous cationic frameworks by crosslinking polyhedral oligomeric silsesquioxane units with N-heterocyclic linkers. *Sci. Rep.* **5**, 11236; doi: 10.1038/srep11236 (2015).

## Supplementary Material

Supplementary Information

## Figures and Tables

**Table 1:** Textural properties and N content of PCIF-1 series with different molar ratios of 4,4′-bpy to ClMePOSS^a^.

**PCIF-1(M*****x*****) series**	**N content [wt%]^b^**	**S_BET_ [m^2^ g^−1^]^c^**	**V_total_ [cm^3^ g^−1^]^d^**	**D_av_ [nm]^e^**
PCIF-1(M2)	0.24	448	0.24	2.16
PCIF-1(M4)	0.64	942	0.76	3.23
PCIF-1(M6)	1.19	696	0.73	4.17
PCIF-1(M8)	1.59	460	0.60	5.24

^a^Reaction conditions: ClMePOSS (0.5 g, 0.62 mmol), molar ratios of 4,4′-bpy to ClMePOSS (*x* = 2, 4, 6, 8), THF (10 mL), 100 ^o^C, 48 h.

^b^N content was measured by CHN elemental analysis.

^c^Brunauer-Emmett-Teller (BET) surface area calculated over the range P/P_0_ = 0.05–0.20.

^d^Total pore volume calculated at P/P_0_ = 0.99.

^e^Average pore size calculated by the BET method.

**Table 2:**
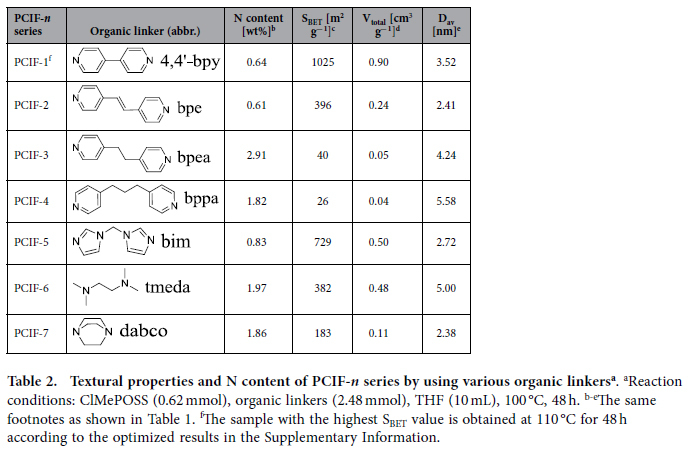
Textural properties and N content of PCIF-*n* series by using various organic linkers^a^.

## References

[b1] Furukawa H., Cordova K. E., O’Keeffe M. & Yaghi O. M. The chemistry and applications of metal-organic frameworks. Science 341, 1230444 (2013). 23990564 10.1126/science.1230444

[b2] Ding S.-Y. & Wang W. Covalent organic frameworks (COFs): from design to applications. Chem. Soc. Rev. 42, 548–568 (2013). 23060270 10.1039/c2cs35072f

[b3] Zhang Y. G. & Riduan S. N. Functional porous organic polymers for heterogeneous catalysis. Chem. Soc. Rev. 41, 2083–2094 (2012) . 22134621 10.1039/c1cs15227k

[b4] Guo J. *et al.* Conjugated organic framework with three-dimensionally ordered stable structure and delocalized π clouds. Nat. Commun. 4, 2736 (2013). 24220603 10.1038/ncomms3736 PMC3868157

[b5] Wu Y. J. *et al.* Framework-substituted lanthanide MCM-22 zeolite: synthesis and characterization. J. Am. Chem. Soc. 132, 17989–17991 (2010). 21141821 10.1021/ja107633j

[b6] Díaz U., Brunel D. & Corma A. Catalysis using multifunctional organosiliceous hybrid materials. Chem. Soc. Rev. 42, 4083–4097 (2013). 23288312 10.1039/c2cs35385g

[b7] Voort P. V. D. *et al.* Periodic mesoporous organosilicas: from simple to complex bridges; a comprehensive overview of functions, morphologies and applications. Chem. Soc. Rev. 42, 3913–3955 (2013). 23081688 10.1039/c2cs35222b

[b8] Cordes D. B., Lickiss P. D. & Rataboul F. Recent developments in the chemistry of cubic polyhedral oligosilsesquioxanes. Chem. Rev. 110, 2081–2173 (2010). 20225901 10.1021/cr900201r

[b9] Tanaka K. & Chujo Y. Advanced functional materials based on polyhedral oligomeric silsesquioxane (POSS). J. Mater. Chem. 22, 1733–1746 (2012).

[b10] Zhang W. & Müller A. H. E. Architecture, self-assembly and properties of well-defined hybrid polymers based on polyhedral oligomeric silsequioxane (POSS). Prog. Polym. Sci. 38, 1121–1162 (2013).

[b11] Morris R. E. Modular materials from zeolite-like building blocks. J. Mater. Chem. 15, 931–938 (2005).

[b12] Zhang L. *et al.* Mesoporous organic-inorganic hybrid materials built using polyhedral oligomeric silsesquioxane blocks. Angew. Chem. Int. Ed. 46, 5003–5006 (2007). 10.1002/anie.200700640 17516599

[b13] Díaz U., García T., Velty A. & Corma A. Synthesis and catalytic properties of hybrid mesoporous materials assembled from polyhedral and bridged silsesquioxane monomers. Chem.-Eur. J. 18, 8659–8672 (2012). 22678926 10.1002/chem.201200170

[b14] Zhang C. *et al.* Highly porous polyhedral silsesquioxane polymers. synthesis and characterization. J. Am. Chem. Soc. 120, 8380–839 (1998).

[b15] Morrison J. J., Love C. J., Manson B. W., Shannon I. J. & Morris R. E. Synthesis of functionalised porous network silsesquioxane polymers. J. Mater. Chem. 12, 3208–3212 (2002).

[b16] Roll M. F., Kampf J. W., Kim Y., Yi E. & Laine R. M. Nano building blocks via iodination of [PhSiO_1.5_]_n_, forming [*p*-I-C_6_H_4_SiO_1.5_]_n_ (n = 8, 10, 12), and a new route to high-surface-area, thermally stable, microporous materials via thermal elimination of I_2_. J. Am. Chem. Soc. 132, 10171–1018 (2010). 20586474 10.1021/ja102453s

[b17] Chaikittisilp W., Sugawara A., Shimojima A. & Okubo T. Hybrid porous materials with high surface area derived from bromophenylethenyl-functionalized cubic siloxane-based building units. Chem.-Eur. J. 16, 6006–6014 (2010). 20391584 10.1002/chem.201000249

[b18] Chaikittisilp W., Sugawara A., Shimojima A. & Okubo T. Microporous hybrid polymer with a certain crystallinity built from functionalized cubic siloxane cages as a singular building unit. Chem. Mater. 22, 4841–4843 (2010).

[b19] Wang D. X. *et al.* Hybrid networks constructed from tetrahedral silicon-centered precursors and cubic POSS-based building blocks via Heck reaction: porosity, gas sorption, and luminescence. J. Mater. Chem. A 1, 13549–13558 (2013).

[b20] Wang D. X., Yang W. Y., Feng S. Y. & Liu H. Z. Constructing hybrid porous polymers from cubic octavinylsilsequioxane and planar halogenated benzene. Polym. Chem. 5, 3634–3642 (2014).

[b21] Chaikittisilp W. *et al.* Porous siloxane organic hybrid with ultrahigh surface area through simultaneous polymerization destruction of functionalized cubic siloxane cages. J. Am. Chem. Soc. 133, 13832–13835 (2011). 21819064 10.1021/ja2046556

[b22] Wu Y. *et al.* Hybrid porous polymers constructed from octavinylsilsesquioxane and benzene via Friedel-Crafts reaction: tunable porosity, gas sorption, and postfunctionalization. J. Mater. Chem. A 2, 2160–2167 (2014).

[b23] Nischang I., Brüggemann O. & Teasdale I. Facile, single-step preparation of versatile, high-surface-area, hierarchically structured hybrid materials. Angew. Chem. Int. Ed. 50, 4592–4596 (2011). 10.1002/anie.201100971 21495148

[b24] Takamizawa S., Akatsuka T. & Ueda T. Gas-conforming transformability of an ionic single-crystal host consisting of discrete charged components. Angew. Chem. Int. Ed. 47, 1689–1692 (2008). 10.1002/anie.200702950 17987635

[b25] Eguchi R., Uchida S. & Mizuno N. Inverse and high CO_2_/C_2_H_2_ sorption selectivity in flexible organic-inorganic ionic crystals. Angew. Chem. Int. Ed. 51, 1635–1639 (2012). 10.1002/anie.201107906 22311812

[b26] Yang S. H. *et al.* Cation-induced kinetic trapping and enhanced hydrogen adsorption in a modulated anionic metal-organic framework. Nat. Chem. 1, 487–493 (2009). 21378916 10.1038/nchem.333

[b27] Fei H. H., Rogow D. L. & Oliver S. R. J. Reversible anion exchange and catalytic properties of two cationic metal-organic frameworks based on Cu(I) and Ag(I). J. Am. Chem. Soc. 132, 7202–7209 (2010). 20426466 10.1021/ja102134c

[b28] Genna D. T., Wong-Foy A. G., Matzger A. J. & Sanford M. S. Heterogenization of homogeneous catalysts in metal-organic frameworks via cation exchange. J. Am. Chem. Soc. 135, 10586–10589 (2013). 23837970 10.1021/ja402577s

[b29] Yu J. C. *et al.* Confinement of pyridinium hemicyanine dye within an anionic metal-organic framework for two-photon-pumped lasing. Nat. Commun. 4, 2719 (2013). 24173352 10.1038/ncomms3719 PMC4089137

[b30] Yuan Y., Sun F. X., Li L. N., Cui P. & Zhu G. S. Porous aromatic frameworks with anion-templated pore apertures serving as polymeric sieves. Nat. Commun. 5, 4260 (2014). 24963967 10.1038/ncomms5260

[b31] Fischer S., Schmidt J., Strauch P. & Thomas A. An anionic microporous polymer network prepared by the polymerization of weakly coordinating anions. Angew. Chem. Int. Ed. 52, 12174–12178 (2013). 10.1002/anie.201303045 24151252

[b32] Fischer S. *et al.* Cationic microporous polymer networks by polymerisation of weakly coordinating cations with CO_2_-storage ability. J. Mater. Chem. A 2, 11825–11829 (2014).

[b33] Neouze M.-A., Kronstein M. & Tielens F. Ionic nanoparticle networks: development and perspectives in the landscape of ionic liquid based materials. Chem. Commun. 50, 10929–10936 (2014). 10.1039/c4cc02419b 24968952

[b34] Karimi B. *et al.* Synthesis and characterization of alkyl-imidazolium-based periodic mesoporous organosilicas: a versatile host for the immobilization of perruthenate (RuO_4_^-^) in the aerobic oxidation of alcohols. Chem.-Eur. J. 18, 13520–13530 (2012). 22945297 10.1002/chem.201200380

[b35] Chen G. J., Zhou Y., Zhao P. P., Long Z. Y. & Wang J. Mesostructured dihydroxy-functionalized guanidinium-based polyoxometalate with enhanced heterogeneous catalytic activity in epoxidation. ChemPlusChem 78, 561–569 (2013).

[b36] Chen G. J. *et al.* Mesoporous polyoxometalate-based ionic hybrid as a triphasic catalyst for oxidation of benzyl alcohol with H_2_O_2_ on water. ACS Appl. Mater. Interfaces 6, 4438–4446 (2014). 24601477 10.1021/am5001757

[b37] Tanaka K., Ishiguro F. & Chujo Y. POSS ionic liquid. J. Am. Chem. Soc. 132, 17649–17651 (2010). 21105729 10.1021/ja105631j

[b38] Hallett J. P. & Welton T. Room-temperature ionic liquids: solvents for synthesis and catalysis. 2. Chem. Rev. 111, 3508–3576 (2011). 21469639 10.1021/cr1003248

[b39] Tan J. L., Ma D. P., Sun X. R., Feng S. Y. & Zhang C. Q. Synthesis and characterization of an octaimidazolium-based polyhedral oligomeric silsesquioxanes ionic liquid by an ion-exchange reaction. Dalton Trans. 42, 4337–4339 (2013). 23223783 10.1039/c2dt32645k

[b40] Leng Y., Liu J., Jiang P. P., & Wang J. POSS-derived mesostructured amphiphilic polyoxometalate-based ionic hybrids as highly efficient epoxidation catalysts. ACS Sustainable Chem. Eng. 3, 170–176 (2015).

[b41] Eaborn C. Some recent studies of the cleavage of carbon-silicon and related bonds. Pure Appl. Chem. 19, 375–388 (1969)

[b42] Krieble R. H. & Elliott J. R. The hydrolytic cleavage of methyl and chloromethyl siloxanes. J. Am. Chem. Soc. 68, 2291–2294 (1946).

[b43] Marciniec B., Dutkiewicz M., Maciejewski H. & Kubicki M. New, effective method of synthesis and structural characterization of octakis(3-chloropropyl)octasilsesquioxane. Organometallics 27, 793–794 (2008).

[b44] Baroncini M. *et al.* Light control of stoichiometry and motion in pseudorotaxanes comprising a cucurbit[7]uril wheel and an azobenzene-bipyridinium axle. Chem.-Eur. J. 20, 10737–10744 (2014). 24931834 10.1002/chem.201402821

[b45] Dawson R., Cooper A. I. & Adams D. J. Nanoporous organic polymer networks. Prog. Polym. Sci. 37, 530–563 (2012).

[b46] Zhang Z. J., Yao Z. Z., Xiang S. C. & Chen B. L. Perspective of microporous metal–organic frameworks for CO_2_ capture and separation. Energy Environ. Sci. 7, 2868–2899 (2014).

[b47] Dawson R., Adams D. J. & Cooper A. I. Chemical tuning of CO_2_ sorption in robust nanoporous organic polymers. Chem. Sci. 2, 1173–1177 (2011).

[b48] Zagorodni A. A. in Ion exchange materials: properties and applications (Elsevier, 2006).

[b49] Zhao P. P., Wang J., Chen G. J., Zhou Y. & Huang J. Phase-transfer hydroxylation of benzene with H_2_O_2_ catalyzed by a nitrile-functionalized pyridinium phosphovanadomolybdate. Catal. Sci. Technol. 3, 1394–1404 (2013).

[b50] Zhou Y., Chen G. J., Long Z. Y. & Wang J. Recent advances in polyoxometalate-based heterogeneous catalytic materials for liquid-phase organic transformations. RSC Adv. 4, 42092–42113 (2014).

[b51] Long Z. Y., Zhou Y., Chen G. J., Ge W. L. & Wang J. C_3_N_4_-H_5_PMo_10_V_2_O_40_: a dual-catalysis system for reductant-free aerobic oxidation of benzene to phenol. Sci. Rep. 4, 3651 (2014). 24413448 10.1038/srep03651 PMC3888967

[b52] Kamata K., Yonehara K., Nakagawa Y., Uehara K. & Mizuno N. Efficient stereo- and regioselective hydroxylation of alkanes catalysed by a bulky polyoxometalate. Nat. Chem. 2, 478–483 (2010). 20489717 10.1038/nchem.648

[b53] Yamaguchi S., Sumimoto S., Ichihashi Y., Nishiyama S. & Tsuruya S. Liquid-phase oxidation of benzene to phenol over V-substituted heteropolyacid catalysts. Ind. Eng. Chem. Res. 44, 1–7 (2005).

[b54] Wang W. T. *et al.* Facile one-pot synthesis of V_x_O_y_@C catalysts using sucrose for the direct hydroxylation of benzene to phenol. Green Chem. 15, 1150–1154 (2013).

[b55] Long Z. Y., Zhou Y., Chen G. J., Zhao P. P. & Wang J. 4,4’-bipyridine-modified molybdovanadophosphoric acid: a reusable heterogeneous catalyst for direct hydroxylation of benzene with O_2_. Chem. Eng. J. 239, 19–25 (2014).

[b56] Long Z. Y. *et al.* Ionic-liquid-functionalized polyoxometalates for heterogeneously catalyzing the aerobic oxidation of benzene to phenol: raising efficacy through specific design. ChemPlusChem 79, 1590–1596 (2014).

[b57] Yang H., Li J., Zhang H. J., Lv Y. & Gao S. Facile synthesis of POM@MOF embedded in SBA-15 as a steady catalyst for the hydroxylation of benzene. Micropor. Mesopor. Mater. 195, 87–91 (2014).

[b58] Alexander M., Khenkin G. & Neumann R. Electron transfer-oxygen transfer oxygenation of sulfides catalyzed by the H_5_PV_2_Mo_10_O_40_ polyoxometalate. J. Am. Chem. Soc. 132, 11446–11448 (2010). 20669975 10.1021/ja105183w

[b59] Mizuno N., Nozaki C., Kiyoto I. & Misono M. Highly efficient utilization of hydrogen peroxide for selective oxygenation of alkanes catalyzed by diiron-substituted polyoxometalate precursor. J. Am. Chem. Soc. 120, 9267–9272 (1998).

[b60] Diez-Barra E., De la Hoz A., Sánchez-Migallón A. & Tejeda J. Phase transfer catalysis without solvent: synthesis of bisazolylalkanes. Heterocycles 34, 1365–1373 (1992).

