# Supplementary material for: Construction of porous cationic frameworks by crosslinking polyhedral oligomeric silsesquioxane units with N-heterocyclic linkers
Source: Sci Rep. 2015 Jun 11;5:11236. doi: 10.1038/srep11236 (PMC4463022; doi:10.1038/srep11236)
Supplement: Supplementary Information [file srep11236-s1.doc]

**Supplementary Information**

**Construction of porous cationic frameworks by crosslinking polyhedral oligomeric silsesquioxane units with N-heterocyclic linkers**

Guojian Chen^†^, Yu Zhou^†^, Xiaochen Wang, Jing Li, Shuang Xue, Yangqing Liu, Qian Wang & Jun Wang*

State Key Laboratory of Materials-Oriented Chemical Engineering, College of Chemistry and Chemical Engineering, Nanjing Tech University, Nanjing 210009, China.

^†^ These authors contributed equally to this work.

* Correspondence and requests for materials should be addressed to J.W.

(email: junwang@njtech.edu.cn).

**Supplementary Experimental Section**

**Synthesis of octakis(3-chloropropyl)silsesquioxane ClPrPOSS.** ClPrPOSS was synthesized by previously reported method with a slight modification. As shown in Supplementary Fig. S1, a solution of 400 mL of dry methanol and 15 mL of concentrated hydrochloric acid was placed in a round-bottomed flask, and then (3-chloropropyl)trimethoxysilane (20 mL) was slowly added into the solution. The reaction mixture within the closely sealed flask, was maintained at temperature of 40^o^C for two weeks until a mass of white crystalline precipitate appeared. The solution was filtered and the crystals were collected, washed several times with methanol, and dried under vacuum. The final product was obtained in 32% yield (4.5 g). Spectroscopic data of ClPrPOSS are as follows (see Supplementary Figs. S6 and 7). ^1^H NMR (500 MHz, CDCl_3_) δ 0.79 (t, 16H, SiCH_2_), 1.86 (qui, 16H, CH_2_), 3.54 ppm (t, 16H, CH_2_Cl). ^13^C NMR (125 MHz, CDCl_3_) δ 8.84 (SiCH_2_), 25.75 (CH_2_), 46.50 ppm (CH_2_Cl).

**Supplementary Figures and Tables**

**Supplementary Figure S1.** Synthesis of octakis(3-chloropropyl)silsesquioxane ClPrPOSS.


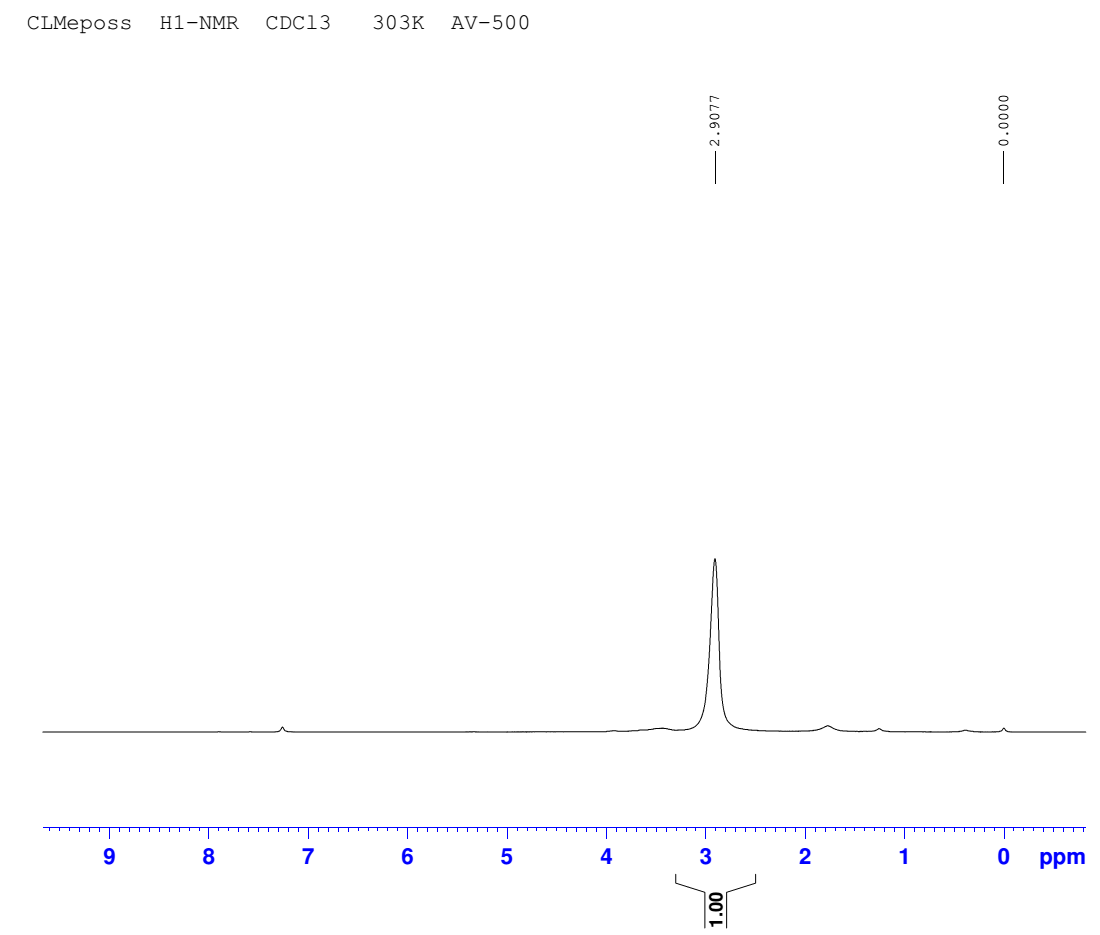


1

**Supplementary Figure S2.** ^1^H NMR of ClMePOSS.


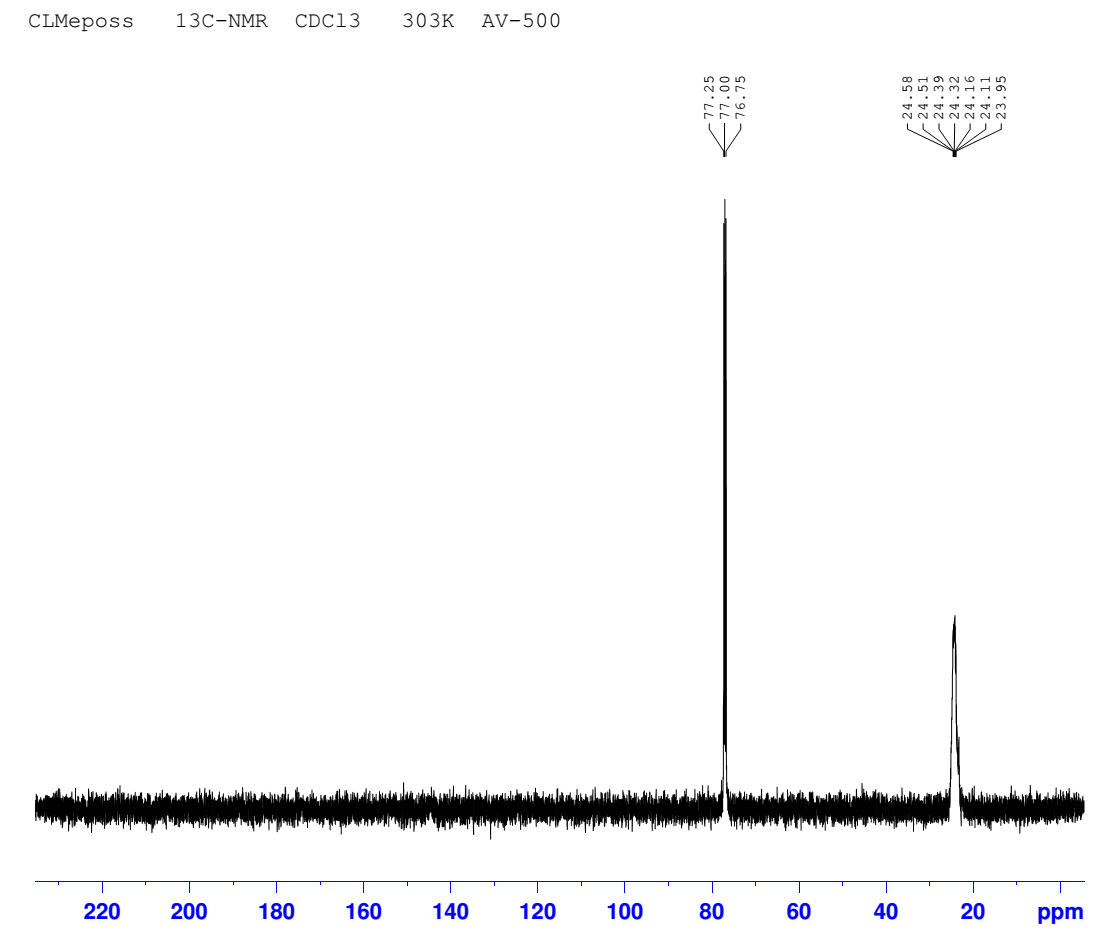


1

**Supplementary Figure S3.** ^13^C NMR of ClMePOSS.


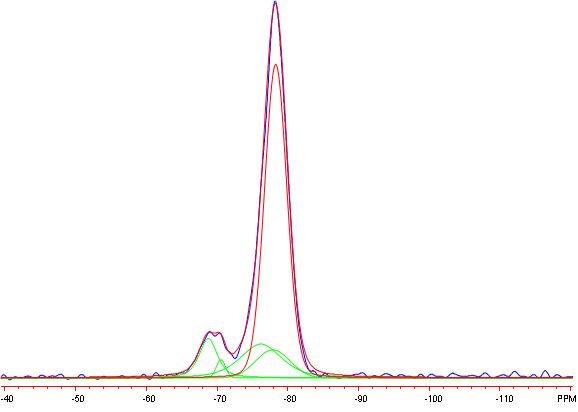


T^3^_α_

T^3^_γ_

T^3^_β_

T^2^_α_

T^2^_β_

b

| T^n^ peaks | ppm |
| --- | --- |
| T^2^_α_ | -68.7 |
| T^2^_β_ | -70.5 |
| T^3^_α_ | -76.2 |
| T^3^_β_ | -77.9 |
| T^3^_γ_ | -78.3 |

**Supplementary Figure S4. (a)** ^29^Si MAS NMR spectra of ClMePOSS and **(b)** its fitting with multiple peaks. A small signal at –69.0 ppm and the main signal at –78.3 ppm for the as-synthesized ClMePOSS are assigned to its T^2^, T^3^ structures with a relatively high T^3^ / (T^2^+T^3^) ratio 0.901, indicating that the new POSS monomer possesses a high percentage (ca. 90%) of T^3^ structure silicons. Fitting the spectrum with multiple peaks reveals three possible T^3^_α_, T^3^_β_, T^3^_γ_ silicon peaks at -76.2, -77.9, -78.3 ppm, respectively, attributable to the T^3^ silicons originated from the minor incompletely condensed POSS units (T^2^_α_, T^2^_β_ silicons) and major completely condensed cubic POSS units (*Macromolecules* **38**, 5088-5097 (2005)). The splitting of the T^3^ signal can be assigned to existence of minor T^2^ silicons in ClMePOSS that disturb the local environments of T^3^ silicons.


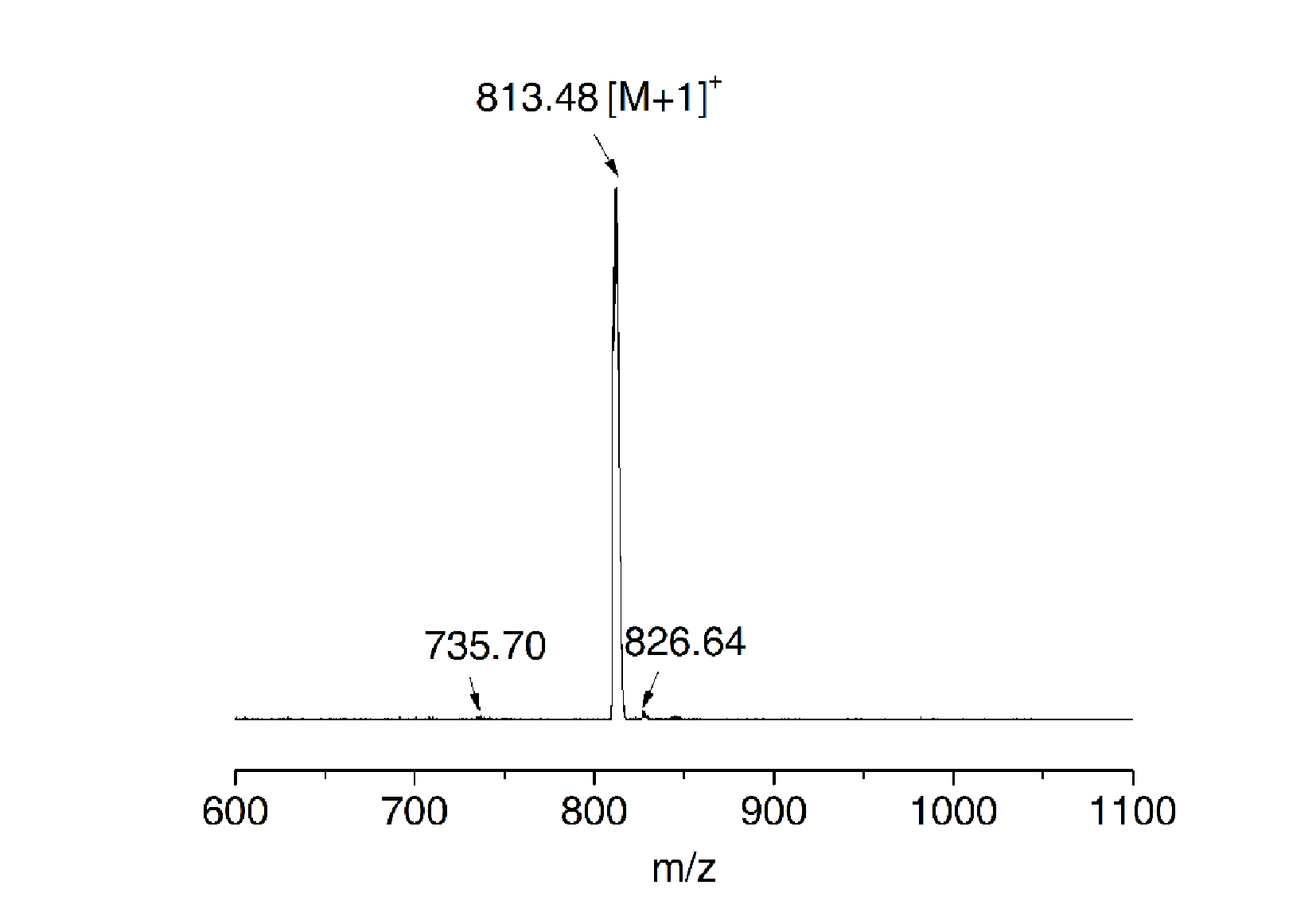


**Supplementary Figure S5.** MALDI-TOF MS spectra of ClMePOSS (DHB matrix, THF as the solvent). The MALDI-TOF MS result gave one sharp strong peak at m/z=813.48, index of T^3^ cubic POSS unit (M_w_=812.48 g mol^-1^). Two very small peaks were observed at m/z=735.70 and 826.64 attributable to incompletely condensed POSS units of trisilanol R_7_Si_7_O_9_(OH)_3_ POSS-triol and R_8_Si_8_O_11_(OH)_2_ POSS-diol respectively (*J. Organomet. Chem*. **693**, 1301-1308 (2008)). The above results indicated that the major POSS units were cubic structure, well in accord with the analysis result of ^29^Si MAS NMR spectrum of ClMePOSS.


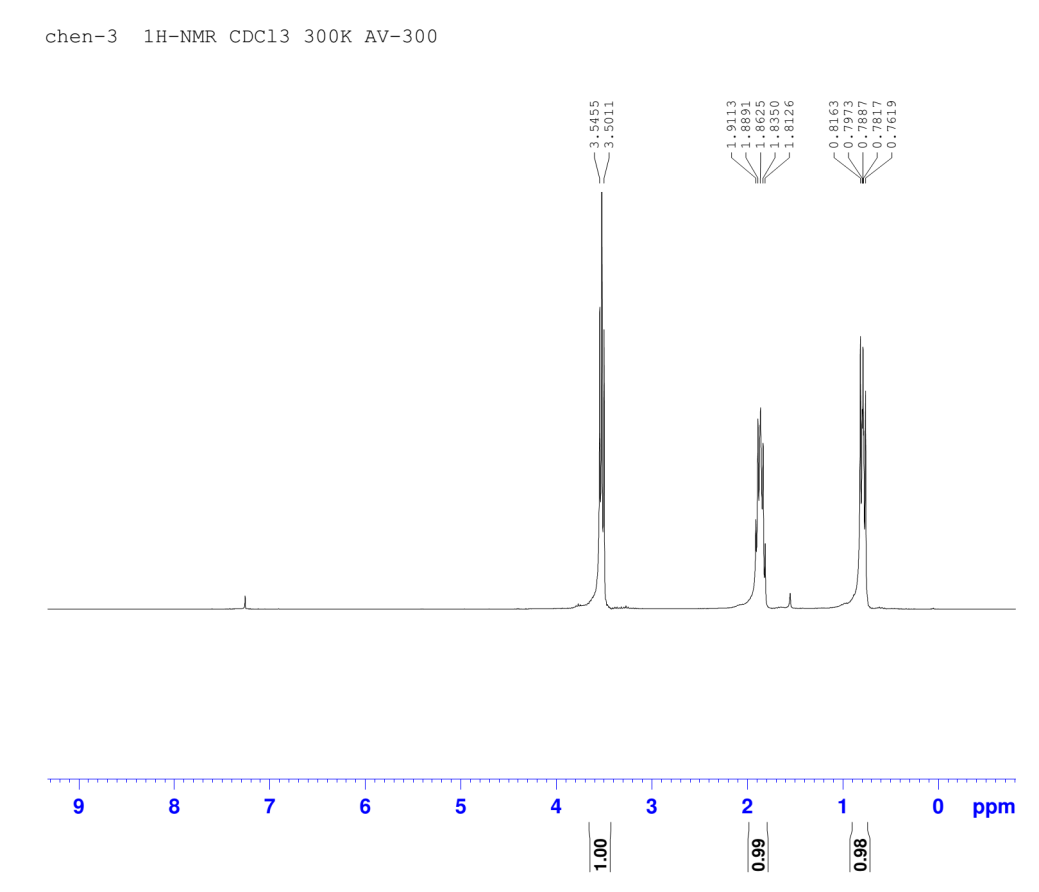


1

2

3

**Supplementary Figure S6.** ^1^H NMR of ClPrPOSS.


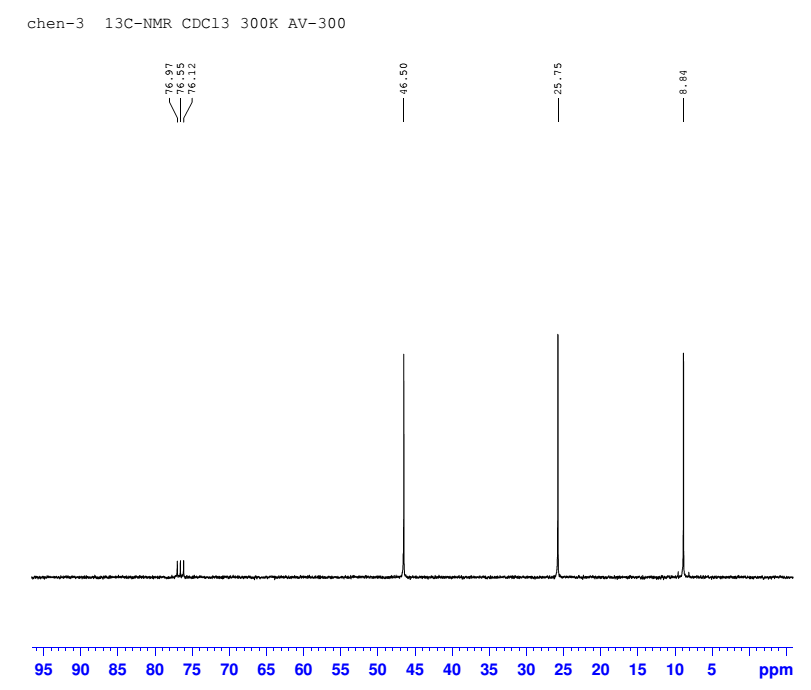


1

2

3

**Supplementary Figure S7.** ^13^C NMR of ClPrPOSS.

**Supplementary Figure S8.** Schematic formation process of Si–C–N bonds, T^n^ and Q^n^ silicons units due to the partial cleavage of Si–C bonds during the synthesis of POSS-based porous cationic framework PCIF-1.

Supplementary Fig. S8 schematically depicts the formation process of Si–C–N bonds, T^n^ and Q^n^ silicons units due to the partial cleavage of Si–C bonds during the synthesis of POSS-based porous cationic framework PCIF-1. The Q^4^-structured POSS units were resulted from the self-condensation of silanols from the formed Q^3^ units under the weak basic environment. Minor Q^2^ structure of silicons may be derived from the cleavage of Si–C in original T^2^ units or the decomposition of siloxane bond in Q^3^ units (not shown in Supplementary Figure 8). Finally, the attack of nucleophilic rigid organic linker and the distortion of POSS cages accelerate the formation of Si–C–N bonds and partial cleavage Si–C bonds to obtain T^n^ and Q^n^ silicons units combined POSS-based porous cationic framework PCIF-1.

**Supplementary Figure S9.** Various N-heterocyclic compounds used for synthesizing water-soluble POSS-ILs.

**Supplementary Figure S10.** BJH pore size distributions of PCIF-1 series of PCIF-1(M2), PCIF-1(M4), PCIF-1(M6), and PCIF-1(M8).

**Supplementary Figure S11.** (**a**) N_2_ adsorption-desorption isotherms and (**b**) BJH pore size distributions of the PCIF-1 network, which was prepared in the toluene solution.

**Supplementary Figure S12.** (**a**) N_2_ adsorption-desorption isotherms and (**b**) NLDFT pore size distributions of PCIF-1(M4) using different reaction time.

**Supplementary Figure S13.** (**a**) N_2_ adsorption-desorption isotherms and (**b**) NLDFT pore size distributions of PCIF-1(M4) series at different reaction temperature.

**Supplementary Figure S14.** (**a**) N_2_ adsorption-desorption isotherms and (**b**) NLDFT pore size distributions of PCIF-1(M4) using different amount of solvent THF.

**Supplementary Figure S15.** Influences of reaction parameters (reaction time, temperature and amount of solvent THF) on the surface area (S_BET_), pore volume (V_total_) and average pore size (D_av_) of PCIF-1(M4) samples.

It was apparently that the surface area, pore volume and average pore size first increased with the reaction time, reaching the maximum value at 48 h, and then decreased with longer reaction time. Similar variations were observed by surveying the influence of reaction temperature and the solvent amount. The results indicate that the surface area, pore volume and average pore size of PCIF materials can be regularly adjusted by those factors, while keeping similar type IV N_2_ sorption isotherms and pore size distributions with sharp peaks centered at ca. 2 nm (Supplementary Figs. S12-S14).

**Supplementary Figure S16.** (**a**) N_2_ adsorption-desorption isotherms and (**b**) NLDFT pore size distributions of PCIF-*n* series samples with *n* corresponding to various organic linkers (2: bpe, 3: bpea, 4: bppa, 5: bim, 6: tmeda, 7: dabco).

**The stability of PCIF-1(M4) in various solvents**.

PCIF-1(M4) was selected as a typical sample to investigate its stability in various solvents. The sample was soaked in water, typical organic solvents (CH_3_OH, CHCl_3_, DMF, and DMSO) for 24 h at the room temperature, and then the samples were recovered by the filtration, washing and drying. All the dry samples were fully characterized by N_2_ sorption, SEM, XRD and FT-IR. N_2_ sorption experiment and textural parameters of the recovered PCIF-1(M4) samples (Supplementary Fig. S17, Supplementary Table S4) demonstrate that the porous structures of PCIF-1(M4) are well retained in spite of the slight changes of BET surface areas, total pore volumes and pore sizes. The above results are further confirmed by the no noticeable change of morphologies and crystalline states (Supplementary Figs. S18 and S19). Besides, no noticeable spectral change were observed in FT-IR spectra (Supplementary Fig. S20), indicating their excellent structural stability in water and organic solvents. In brief, the PCIF materials possess well stability in water and common organic solvents.

**Supplementary Figure S17.** (**a**) N_2_ adsorption-desorption isotherms and (**b**) NLDFT pore size distributions of PCIF-1(M4) after soaked in H_2_O or various organic solvents (CH_3_OH, CHCl_3_, DMF, and DMSO). The asterisk (*) before solvents represents the sample of PCIF-1(M4) treated in this solvent.


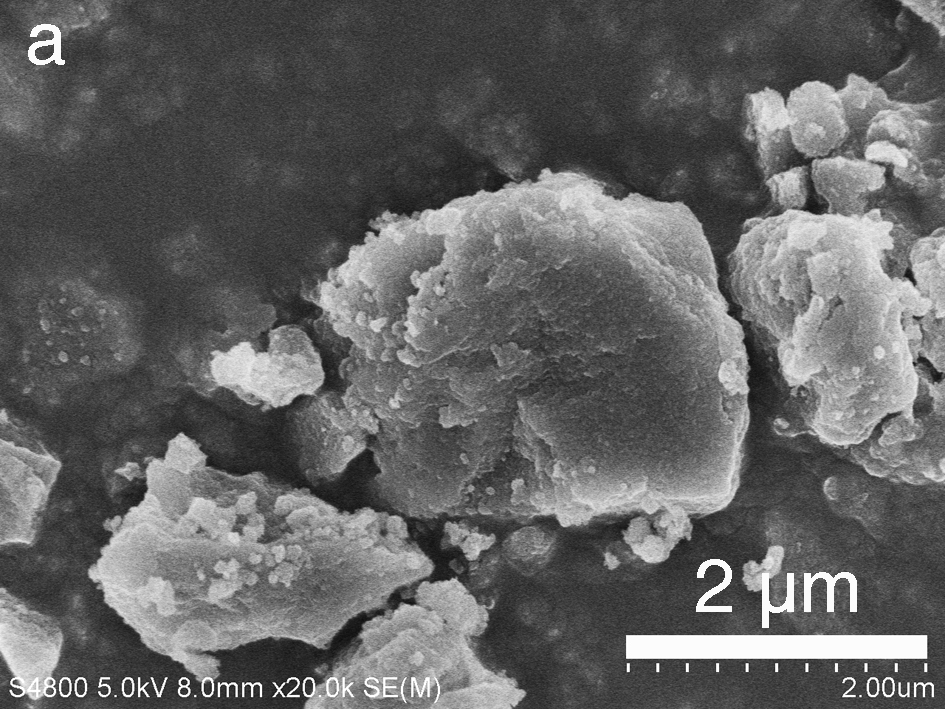

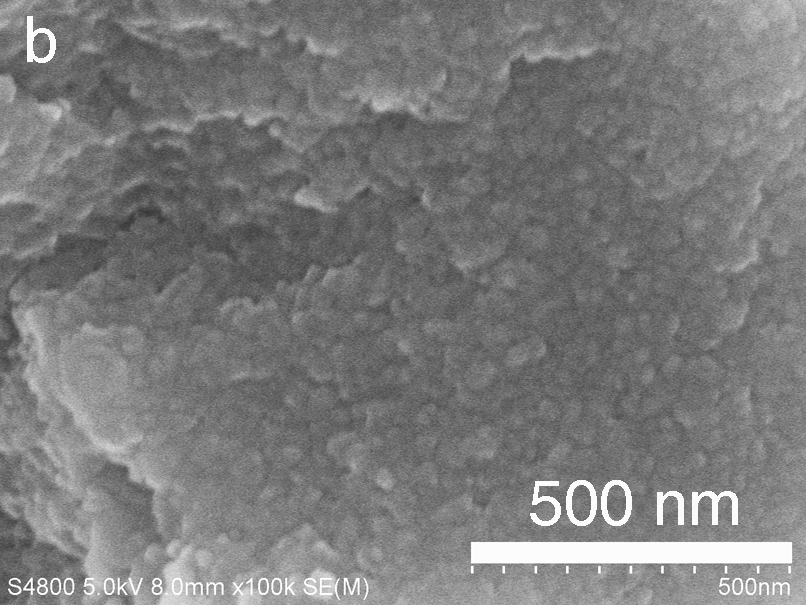


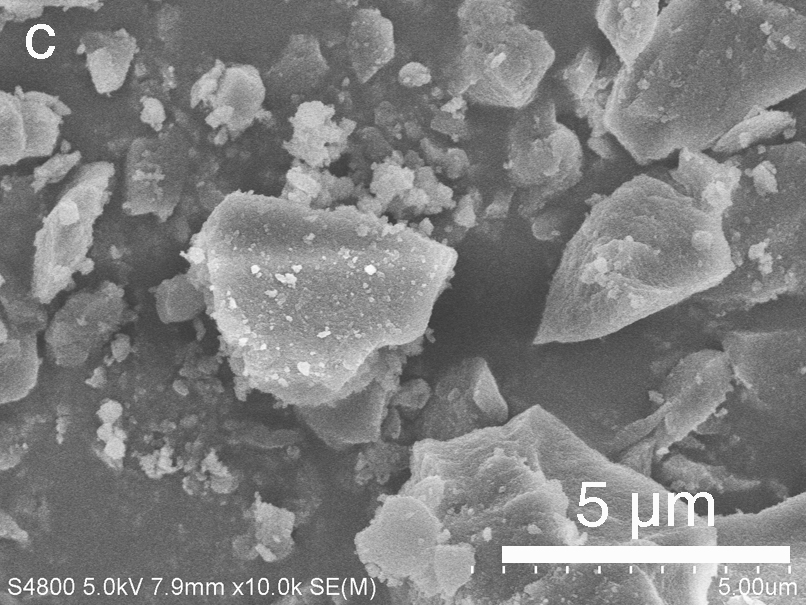

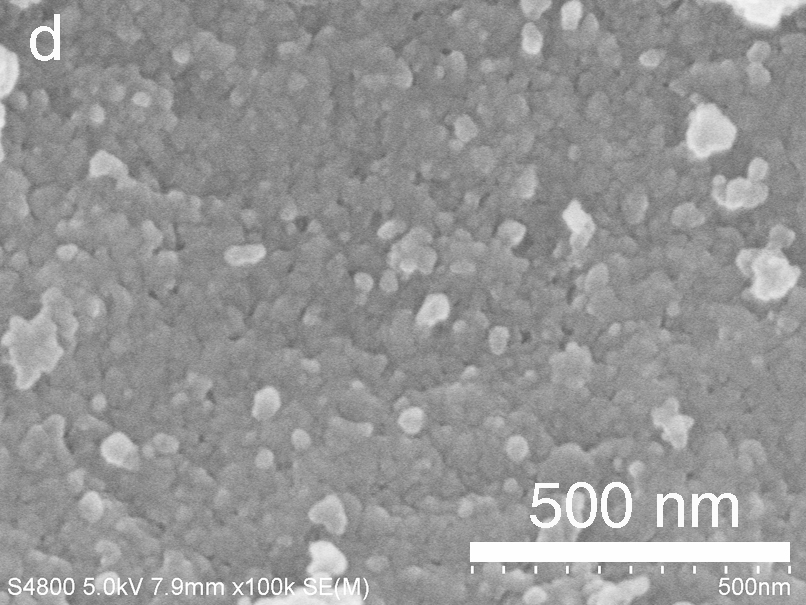


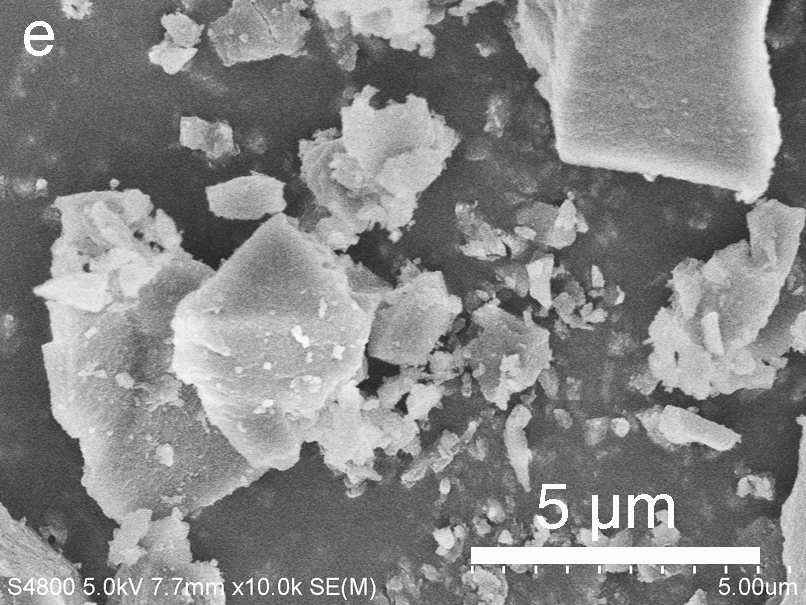

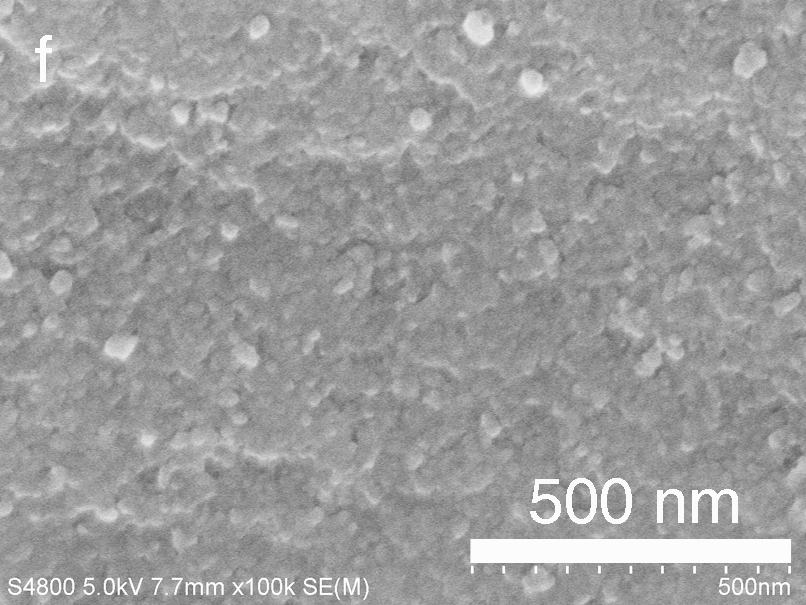


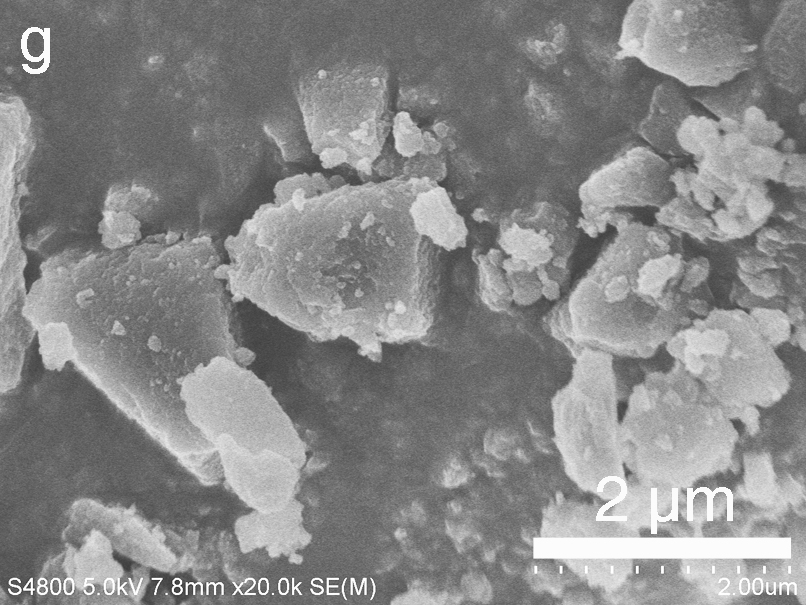

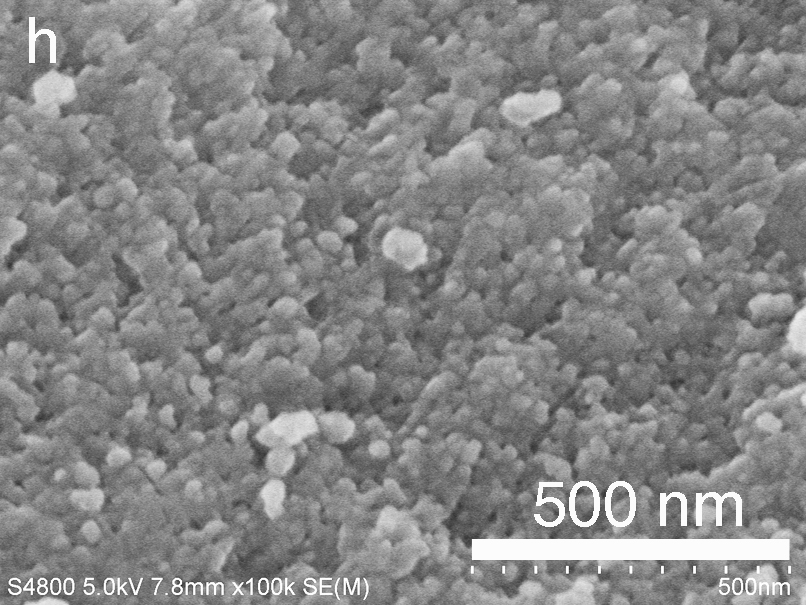


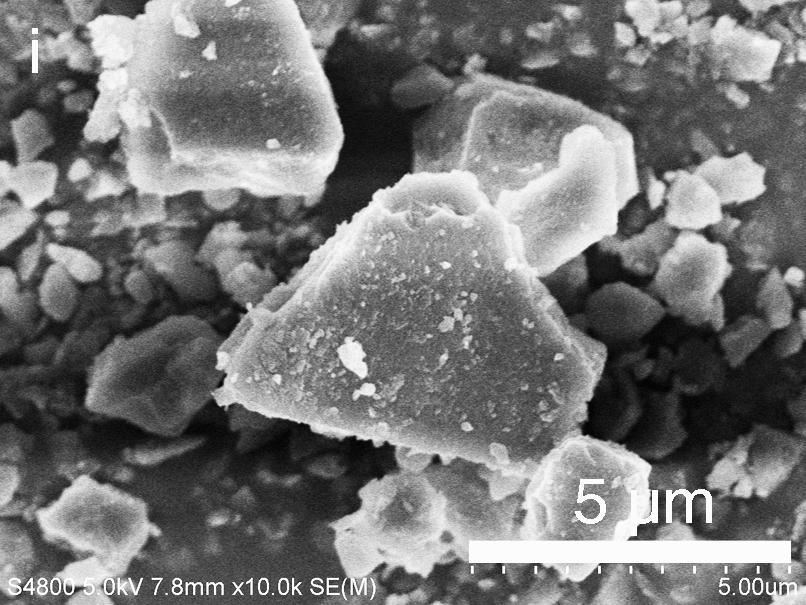

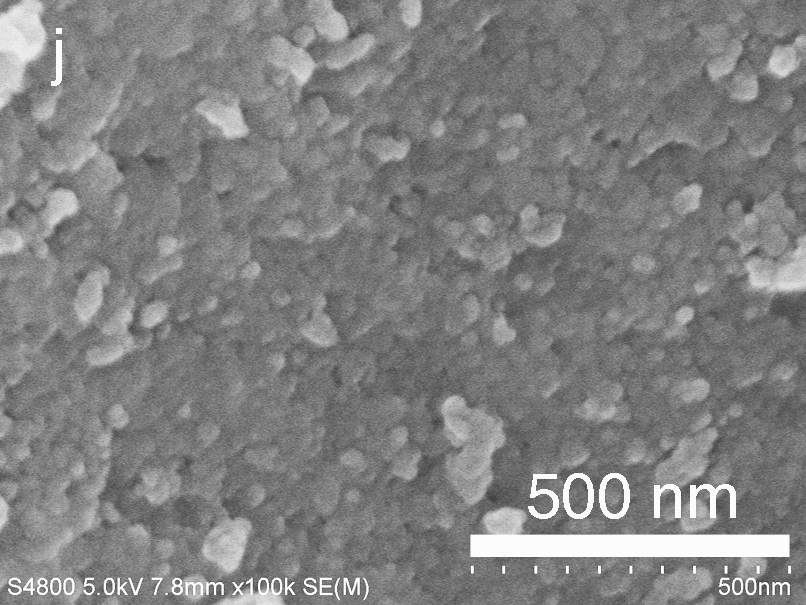


**Supplementary Figure S18.** (**a**) SEM images of PCIF-1(M4) after treated in (a, b) H_2_O, (c, d) CH_3_OH, (e, f) CHCl_3_, (g, h) DMF, and (i, j) DMSO.

**Supplementary Figure S19.** XRD patterns of PCIF-1(M4) after treated in H_2_O or various organic solvents (CH_3_OH, CHCl_3_, DMF, and DMSO). The asterisk (*) before solvents represents the sample of PCIF-1(M4) soaked in this solvent.

**Supplementary Figure S20.** FT-IR spectra of PCIF-1(M4) and the ones after treated in H_2_O or various organic solvents (CH_3_OH, CHCl_3_, DMF, and DMSO). The asterisk (*) before solvents represents the sample of PCIF-1(M4) soaked in this solvent.

**Supplementary Figure S21.** CO_2_ adsorption isotherms of PCIF-1 series (**a**) PCIF-1(M4) at 273 K, 1.0 bar; (**b**) PCIF-1(M4), (**c**) PCIF-1(M6), and (**d**) PCIF-1(M8) at 298 K, 1.0 bar.


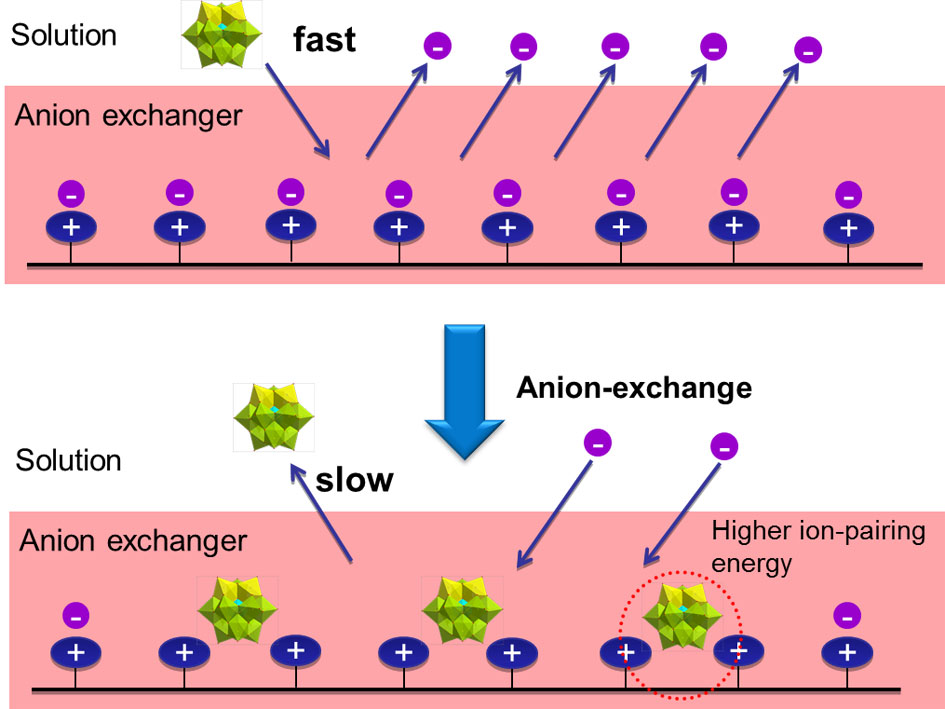


**Supplementary Figure S22.** Schematic image for anion exchange process of very bulky PMoV anions to replace the small Cl ions. Referring to the book of “Ion Exchange Materials: Properties and Applications” by Andrei A. Zagorodni.

**Supplementary Figure S23.** TGA curves of PCIF-1(M8) and PMoV@PCIF-1 under air atmosphere.


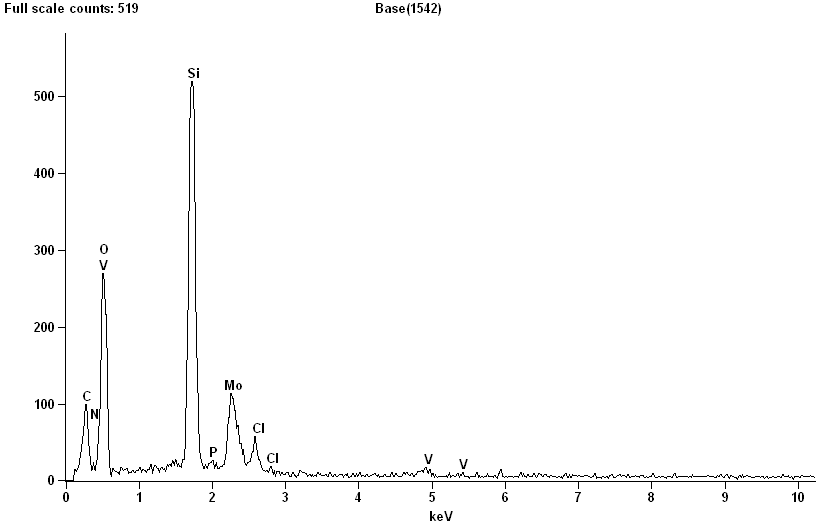


**Cl: 1.81 wt%**

**Supplementary Figure S24.** Energy-dispersive X-ray spectrometry (EDS) spectrum and element distributions of PMoV@PCIF-1.

**Supplementary Figure S25.** High resolution XPS spectra of PMoV@PCIF-1. **(a)** Survey, **(b)** C1s, **(c)** O1s, **(d)** Si2p, **(e)** Cl2p, **(f)** P2p, **(g)** Mo3d, **(h)** Mo3p and N1s, and **(i)** V2p.

**Supplementary Figure S26.** (**a**) N_2_ adsorption-desorption isotherms and (**b**) BJH pore size distributions of PCIF-1(M8) and PMoV@PCIF-1.

**Supplementary Table S1.** Textural properties of PCIF-1(M4) influenced by different reaction time.^a^

| Time (h) | S_BET_ [m^2^ g^-1^]^b^ | V_p_ [cm^3^ g^-1^]^c^ | D_av_ [nm]^d^ |
| --- | --- | --- | --- |
| 12 | 772 | 0.48 | 2.42 |
| 24 | 811 | 0.55 | 2.63 |
| 36 | 839 | 0.57 | 2.80 |
| 48 | 892 | 0.72 | 3.25 |
| 72 | 633 | 0.47 | 2.95 |

[a] Reaction conditions: ClMePOSS (0.5 g, 0.62 mmol), 4,4'-bpy (0.39 g, 2.48 mmol), THF (10 mL), 100 ^o^C, 12~72 h. [b] BET surface area calculated over the range P/P_0_=0.05~0.20. [c] Total pore volume calculated at P/P_0_=0.99. [d] Average pore size calculated by the BET method.

**Supplementary Table S2.** Textural properties of PCIF-1(M4) influenced by different reaction temperature.^a^

| Temperature (^o^C) | S_BET_ [m^2^ g^-1^]^b^ | V_p_ [cm^3^ g^-1^]^c^ | D_av_ [nm]^d^ |
| --- | --- | --- | --- |
| 80 | 500 | 0.26 | 2.07 |
| 90 | 863 | 0.55 | 2.53 |
| 100 | 942 | 0.76 | 3.23 |
| 110 | 1025 | 0.90 | 3.52 |
| 120 | 865 | 0.70 | 3.22 |

[a] Reaction conditions: ClMePOSS (0.5 g, 0.62 mmol), 4,4'-bpy (0.39 g, 2.48 mmol), THF (10 mL), 80~120 ^o^C, 48 h. [b] BET surface area calculated over the range P/P_0_=0.05~0.20. [c] Total pore volume calculated at P/P_0_=0.99. [d] Average pore size calculated by the BET method.

**Supplementary Table S3.** Textural properties of PCIF-1(M4) with different amount of solvent THF.^a^

| THF volume (mL) | S_BET_ [m^2^ g^-1^]^b^ | V_p_ [cm^3^ g^-1^]^c^ | D_av_ [nm]^d^ |
| --- | --- | --- | --- |
| 5 | 763 | 0.62 | 3.27 |
| 10 | 892 | 0.72 | 3.25 |
| 15 | 886 | 0.65 | 2.93 |
| 20 | 843 | 0.63 | 3.00 |

[a] Reaction conditions: ClMePOSS (0.5 g, 0.62 mmol), 4,4'-bpy (0.39 g, 2.48 mmol), THF (5~10 mL), 100 ^o^C, 48 h. [b] BET surface area calculated over the range P/P_0_=0.05~0.20. [c] Total pore volume calculated at P/P_0_=0.99. [d] Average pore size calculated by the BET method.

**Supplementary Table S4.** Textural properties of PCIF-1(M4) after treated in H2O or various organic solvents.a

| Soaked in solvents | S_BET_ [m^2^ g^-1^]^b^ | V_p_ [cm^3^ g^-1^]^c^ | D_av_ [nm]^d^ |
| --- | --- | --- | --- |
| H_2_O | 912 | 0.73 | 3.20 |
| CH_3_OH | 886 | 0.68 | 3.06 |
| CHCl_3_ | 843 | 0.59 | 2.96 |
| DMF | 856 | 0.57 | 2.68 |
| DMSO | 866 | 0.65 | 2.99 |

[a] The sample PCIF-1(M4) with the surface area of 942 m^2^ g^-1^ and pore volume of 0.76 cm^3^ g^-1^ was soaked into various solvents for 24 h at the room temperature, and then was collected by filtration, washing and drying for further characterizations. [b] BET surface area calculated over the range P/P_0_=0.05~0.20. [c] Total pore volume calculated at P/P_0_=0.99. [d] Average pore size calculated by the BET method.

**Supplementary Table S5.** Hydroxylation of benzene to phenol over various catalysts using O_2_ as the oxidant.

| Entry | Catalyst | Yield of phenol (%) |
| --- | --- | --- |
| 1 | No catalyst, no ascorbic acid | Not detected |
| 2 | No catalyst, with ascorbic acid | 2.3 |
| 3 | PCIF-1(M8) (0.25 g) | 2.2 |
| 4 | PMoV (0.035 g) | 4.6 |
| 5 | PMoV@PCIF-1 (0.30 g) | 12.0 |

Reaction condition: 0.8 g ascorbic acid, 2.0 mL benzene, 25 mL aqueous acetic acid solution (50 vol%), 2.0 MPa O_2_, 100 ^o^C, 10 h.

**Supplementary Table S6.** Comparison of catalyst recycling performances of this work with various published heterogeneous catalysts for hydroxylation of benzene with O_2_.

| Catalyst | Phenol yield (%) | Reference |
| --- | --- | --- |
| V/SiO_2_ | 3.7 (one-run) | *J. Chem. Soc. Perkin Trans*. **2**, 847-853 (1999). |
| V/Al_2_O_3_ | 8.2 (one-run)^a^ | *J. Mol. Catal. A: Chem.* **184**, 215–222 (2002). |
| VO_x_/CuSBA-15 | 27 (one-run)^b^ | *Appl. Catal. A* **328**, 150-155 (2007). |
| V-SBA-16 | 30.4 (one-run) | *Catal. Lett.* **142**, 619–626 (2012). |
| V_x_O_y_@C | 9.4/7.0/2.2^c^ | *Green Chem.* **15**, 1150-1154 (2013). |
| Cu/Al_2_O_3_ | 2.5/1.4/0.9 | *J. Mol. Catal. A: Chem.* **208**, 203-211 (2004). |
| LaO_x_/HZSM-5^d^ | 4.2/3.3/3.6 | *Top. Catal*. **47**, 98-100 (2008). |
| CsPMoV_2_ | 7.2/5.1/1.3 | *Ind. Eng. Chem. Res*. **44**, 1-7 (2005). |
| bipy_2_PMoV_1_^e^ | 7.8/5.5/4.2/3.7 | *Chem. Eng. J.* **239**, 19-25 (2014). |
| PMo_9_V_3_@HKUST-1^f^ | 7.37/7.04/6.40/6.35 | *Catal. Commun*. **35**, 101-104 (2013). |
| POM@MOF@SBA-15 | 6.0/6.0/5.8/5.9 | *Micropor. Mesopor. Mater.* **195**, 87-91 (2014) |
| **PMoV@PCIF-1** | **12.0/9.3/8.4/6.8** | **This work** |

[a] 37.6 % V leaching observed. [b] Vanadium was dissolved obviously into the liquid phase. [c] The supported vanadium catalysts suffered from the vast leaching of vanadium. [d] HZSM-5: hydrogen form of ZSM-5 zeolite. [e] bipy: 4,4'-bipyridine. [f] HKUST-1: a stable metal-organic framework (MOF).

In order to make a detail comparison, Supplementary Table S6 summarizes the initial phenol yield and recycling performances of the typical previous reported heterogeneous catalysts for hydroxylation of benzene with O_2_. Up to now, only very few heterogeneous catalysts have been reported so far, and they rarely exhibited both high phenol yield and well reusability. VO_x_/CuSBA-15 and V-SBA-16 gave the highest phenol yield of more than 20%, but these catalysts could not be recycled (*Appl. Catal. A* **328**, 150-155 (2007); *Catal. Lett.* **142**, 619–626 (2012)). The recently reported POM catalysts including bipy_2_PMoV_1_, PMo_9_V_3_@HKUST-1 and POM@MOF@SBA-15 gave the improved catalytic recycle performances, but the phenol yields were modest (*Chem. Eng. J.* **239**, 19-25 (2014); *Catal. Commun*. **35**, 101-104 (2013); *Microporous Mesoporous Mater.* **195**, 87-91 (2014)). By comparison, the four-run cycle performance of 12.0/9.3/8.4/6.8% over the catalyst PMoV@PCIF-1 is superior to the previously reported heterogeneous catalytic systems, presenting both relative high phenol yield and well cycle performance at the current research stage. More importantly, to the best of our knowledge, the TON value (136) is much higher than all the previous V-POM-based catalysts for homogeneous or heterogeneous aerobic oxidation of benzene to phenol, further illustrating the high activity of the obtained PMoV@PCIF-1. In a word, the presented catalyst PMoV@PCIF-1 provided both relative high initial phenol yield and well cycle performance.
